# Immunomodulatory mechanisms of gut microbiota-derived metabolites in non-small cell lung cancer: a gut–lung axis perspective

**DOI:** 10.3389/fimmu.2026.1999117

**Published:** 2026-10-30

**Authors:** Shumin Ye, Tian Tian, Junkang Huangfu, Huimin Liu, Peiyan Liu, Jun Zhou

**Affiliations:** 1 Changzhou Medical Center, Nanjing Medical University, Changzhou, China; 2 Soochow University, Suzhou, China; 3 Department of Pulmonary and Critical Care Medicine, The First People’s Hospital of Changzhou, Changzhou, China

**Keywords:** gut microbiota, gut–lung axis, immunotherapy, metabolites, non-small cell lung cancer

## Abstract

Immunotherapy has emerged as a crucial component in the treatment of non-small cell lung cancer (NSCLC). Nevertheless, interindividual variability in efficacy, along with both primary and acquired resistance, as well as immune-related adverse events, continue to limit the benefits for patients. The gut microbiota and its associated metabolites are believed to play a significant role in pulmonary immune regulation via the gut–lung axis, with alterations in this system being associated with NSCLC development and progression, as well as the outcomes of immunotherapy. This review summarizes the key features of gut microbial and metabolic profiles in patients with NSCLC, focusing particularly on the mechanisms by which short-chain fatty acids, bile acids, gut microbiota-associated Trp metabolites, and other microbial metabolites may influence the tumor immune microenvironment and responses to immunotherapy. Furthermore, we consider their potential value in supporting NSCLC diagnosis, prognostic assessment, and treatment-response prediction. Therapies aimed at targeting metabolic pathways, fecal microbiota transplantation, dietary interventions, and microbiome-based preparations present additional avenues for enhancing the efficacy of NSCLC immunotherapy and managing treatment-related toxicity. However, current evidence primarily stems from small clinical studies and animal models, while methodological discrepancies and limited causal evidence impede clinical translation. Future research that integrates longitudinal multi-omics analyses, metabolic tracing, and controlled clinical trials is essential to elucidate the clinical significance of the gut microbiota-metabolite axis in NSCLC immunotherapy and precision medicine.

## 1 Introduction

Lung cancer represents a significant global health issue, classified as one of the most severe malignant tumors. Recent statistics from Global Cancer indicate that there are close to 2.6 million newly diagnosed lung cancer cases across the globe, resulting in about 1.9 million fatalities; it holds the top position among all malignant tumors regarding both incidence and mortality rates (1). Non-small cell lung cancer (NSCLC) is the predominant histological subtype, accounting for approximately 85% of lung cancer cases (2). Treatment of NSCLC has gradually shifted from a traditional model centered on surgery, radiotherapy and chemotherapy towards a multimodal and individualized approach that integrates these modalities with targeted therapy and immunotherapy according to disease stage, histological subtype and molecular characteristics (2). Over the past decade, there has been a significant increase in the application of immune checkpoint inhibitors (ICIs) that target programmed death receptor-1 (PD-1), programmed death ligand-1 (PD-L1) and cytotoxic T-lymphocyte-associated antigen-4 (CTLA-4) for cancer treatment, improving long-term survival rates for certain NSCLC patients (3, 4). However, patient responses to immunotherapy vary; a considerable subset of patients exhibits primary resistance, whereas others develop acquired resistance after an initial response, and overall survival benefits remain relatively limited in some patients (5). At the same time, immune-related adverse events (irAEs) may affect treatment safety, tolerability and continuity; in severe cases, they may even lead to discontinuation of therapy (6, 7). Hence, it is essential to further identify the key elements that affect the effectiveness and toxicity of immunotherapy in NSCLC, as well as to investigate safe and efficient personalized sensitization strategies.

In the exploration of sensitization strategies, attention has been drawn to the gut microbiota because of its overarching role in regulating immune homeostasis. The gut microbiota is not only involved in the formation and maturation of the host immune system but also able to influence remotely pulmonary immune responses through gut-derived metabolites (8), making the gut–lung axis a key entry point for interpreting variations in immunotherapy efficacy and identifying sensitization targets. The gut–lung axis is an important anatomical and physiological foundation for immune regulation and serves as a network for bidirectional communication between the lungs and the gut. This cross-organ communication system relies on the transmission microbial metabolites through the systemic circulation and the trafficking of immune cells between anatomically distant tissues (8, 9). It not only allows gut signals to ascend and regulate pulmonary immune homeostasis but also enables pulmonary inflammation or infection to descend and influence gut barrier function and microbiota composition, forming a dynamic feedback loop (10). The digestive and respiratory tracts share an endodermal origin and similar mucosal immunological organizational characteristics; this common origin provides the structural basis for bidirectional gut–lung regulation (11).

Previous reviews have summarized that dysbiosis of the gut microbiota is common in patients with lung cancer, and microbial composition may be associated with clinical responses to ICIs, suggesting that the gut microenvironment may affect anti-tumor responses through metabolite-mediated systemic immune regulation (12–14). Clinical studies suggest that responders tend to harbor a more diverse microbiota, a greater abundance of beneficial gut bacteria, higher short-chain fatty acid (SCFA) levels, whereas non-responders may show lower diversity and fewer such bacteria (15–17). For instance, in patients with advanced NSCLC receiving ICIs, Jin et al. reported that baseline diversity of the gut microbiota was positively correlated with both response to immunotherapy and peripheral immune cell activity (15). These findings collectively point to a core concept: the gut microbiota and its associated metabolites may be linked to variations in the response to immunotherapy. Viewed through the lens of the gut–lung axis, a systematic analysis of the potential immunoregulatory roles of gut microbiota-derived metabolites promises to provide new targets and strategies for addressing challenges in NSCLC immunotherapy. Unlike existing reviews that have primarily focused on gut microbiota composition, this paper centers on gut microbiota-derived metabolites as potential functional mediators. Starting from metabolite-mediated immunoregulatory mechanisms, it simultaneously examines their bidirectional translational potential in developing diagnostic biomarkers for NSCLC while also improving the effectiveness of immunotherapy. Given that current evidence predominantly concerns NSCLC, this review focuses primarily on this subtype; evidence for SCLC is scarce and is discussed only in limitations.

## 2 Studies on the structure and metabolomic characteristics of patients’ gut microbiota

### 2.1 Changes in gut microbiota composition during the development of NSCLC

The onset and progression of lung cancer involve not only local changes in the pulmonary microbiome but are also accompanied by stage-specific alterations in the distal gut microbiota. In healthy adults, *Bacillota* and *Bacteroidota* are generally the predominant phyla in the gut microbiota, while *Actinomycetota* and *Pseudomonadota* are also commonly detected (18, 19), although their relative abundances may vary among individuals and populations. Compared with healthy controls, lung cancer cohorts have shown alterations in gut microbial composition, including a *Firmicutes/Bacteroidetes* (F/B) ratio (corresponding to *Bacillota/Bacteroidota* under updated nomenclature) in some cohorts (20, 21), increased abundance of potentially pathogenic taxa such as *Pseudomonadota* and *Enterobacteriaceae* (20, 21), and reduced abundance of potentially beneficial bacteria such as *Bifidobacterium* (20, 22).

Various clinical features, including tumor-node-metastasis (TNM) staging, distant metastasis, histological subtypes and epidermal growth factor receptor (EGFR) mutation status, are accompanied by characteristic alterations in the microbiota, suggesting that dysbiosis is closely linked to the onset and progression of lung cancer, particularly NSCLC. With lung cancer advancing from initial to later stages, the gut microbiota gradually exhibits a trend towards a ‘decline in protective taxa’ and an ‘enrichment of disease-associated bacteria’. *Veillonellaceae, Dialister* and the butyrate-producing bacterium *Roseburia*, which are associated with gut metabolic homeostasis, show a gradual decline from healthy individuals, through benign nodules and early-stage lung cancer, to advanced-stage lung cancer (23); conversely, the disease-associated bacterium *Bacteroides coprocola* gradually increases with disease progression and is significantly enriched in advanced-stage NSCLC (23). Clinical observational evidence on TNM staging has further revealed that inflammation- or progression-associated bacterial communities, such as *Prevotella*, *Ruminococcus gnavus* and *Rodentibacter*, are associated with T4, N3/M or stage III NSCLC (24). In cases of distant metastasis, particularly central nervous system metastasis, patients with brain metastases from NSCLC exhibit reduced levels of SCFA-producing bacteria—such as *Faecalibacterium*, *Bifidobacterium* and *Butyricicoccus*—as well as reduced levels of acetic acid and butyric acid (21); in contrast, an elevated F/B ratio, accompanied by abnormalities in the metabolism of tryptophan (Trp), nicotinic acid and nicotinamide, was observed in a nude mouse model of NSCLC leptomeningeal metastasis (25).

Qin et al. conducted a stratified analysis based on the degree of pathological invasion in lung adenocarcinoma (LUAD). The findings revealed that the structure of the microbial community in both the atypical adenomatous hyperplasia and the adenocarcinoma *in situ* groups bore a closer resemblance to that observed in healthy controls. In contrast, the microbial changes in the micro-invasive and invasive adenocarcinoma groups were more pronounced, with decreases in *Bacillota* and the F/B ratio observed in these groups. Concurrently, there was a decline in genera such as *Faecalibacterium* and *Roseburia*, both of which produce SCFAs or possess anti-inflammatory properties—accompanied by an enrichment of inflammatory or tumor-associated bacteria such as *Klebsiella.* These findings suggest that gut microbiota dysbiosis may be more pronounced in early-stage LUAD with greater pathological invasion (26). Furthermore, significant microbial heterogeneity also exists within advanced NSCLC. Patients with EGFR wild-type tumors exhibit relatively higher levels of *Pseudomonadota*, while those with EGFR-mutant tumors have higher levels of *Bacteroidota*, *Bacillota* and α-diversity. Following treatment, particularly after chemotherapy, the gut microbiota composition experiences more significant alterations in patients with EGFR wild-type tumors (27).

Diagnostic models constructed based on differential microbiota have demonstrated promising discriminatory performance in the studied cohorts. For example, Zheng et al. developed a 13-OTU-based model for identifying early-stage NSCLC (28). Through a multi-cohort meta-analysis of lung cancer populations with different histological subtypes and stages, Han et al. further found that microbial biomarkers such as *Enterococcus*, *Lactobacillus* and *Escherichia* possess certain discriminatory value in both gut and respiratory tract samples, with the corresponding models achieving AUCs of 0.81 and 0.90, respectively (29). These results indicate that lung cancer-associated microbiota dysbiosis is associated with disease status and may have potential as a non-invasive auxiliary screening marker. However, its clinical application still requires validation through larger-scale, multicenter, prospective cohort studies. Moreover, it should be noted that changes in the F/B ratio are not consistent across studies and should not be considered a reliable standalone indicator of gut microbiota dysbiosis (**Figure 1**).

**Figure 1:**
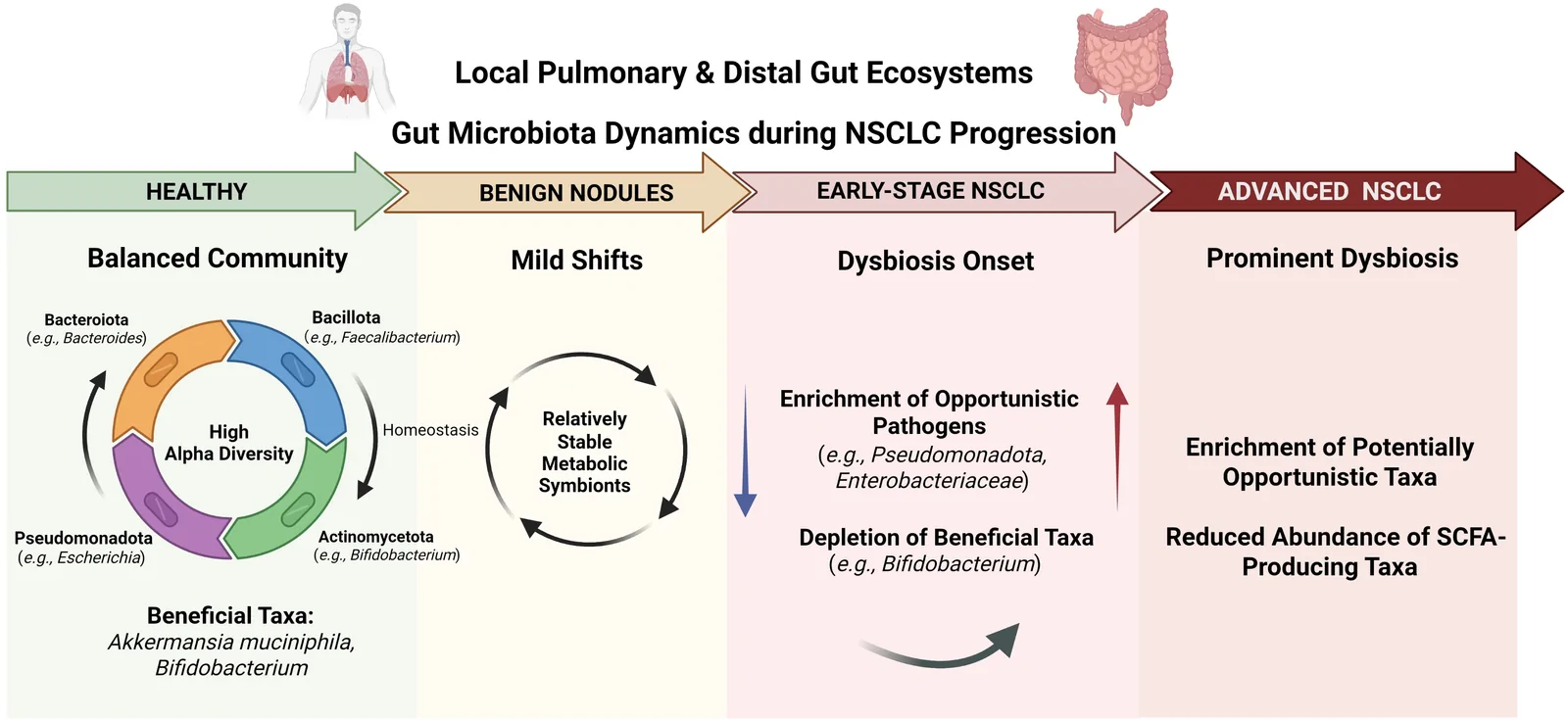
Dynamic shifts in gut microbiota composition during NSCLC progression: the schematic illustrates reported changes in gut microbial composition from healthy individuals and patients with benign pulmonary nodules to those with early-stage and advanced NSCLC. These alterations may involve changes in microbial diversity, taxonomic composition, including potentially beneficial and opportunistic taxa, and the *Bacillota*/*Bacteroidota* (F/B) ratio. However, changes in the F/B ratio are not consistent across studies and should not be considered a universally reliable indicator of gut dysbiosis. The illustrated patterns represent reported associations rather than a uniform progression across all patients. Created with BioRender.com.

### 2.2 Identification and classification of differential metabolites in the gut microbiota of patients with NSCLC

The gut microbiota-associated metabolic network comprises three categories: metabolites directly produced by the gut microbiota, host–microbiota co-metabolites, and microbiota-regulated host metabolites together with dietary precursors. The gut microbiota converts dietary and host-derived substrates into directly microbiota-derived metabolites, including SCFAs and microbial indole derivatives. In addition, host-synthesized primary bile acids are converted by gut bacteria into secondary bile acids (SBAs), representing host–microbiota co-metabolites. Host tryptophan metabolism via IDO1, IDO2 and TDO2 is indirectly regulated by the microbiota. However, Trp itself is a dietary essential amino acid, rather than a metabolite produced directly by the gut microbiota. These metabolites and precursors mediate bidirectional communication between the gut and lung tissue via the hematolymphatic circulation; they may act directly on surface receptors of pulmonary immune cells (8), or activate gut immune cells that subsequently migrate to the lungs (30), thereby collectively constituting the gut–lung axis. For clarity, the following sections are organized according to the major chemical classes—SCFAs, SBAs and microbiota-derived Trp metabolites.

Fecal and serum levels of microbiota-derived metabolites are dysregulated in patients with NSCLC. SCFAs, comprising butyrate, acetate and propionate, show alterations in lung cancer patients, primarily manifested as direct decreases or increases in SCFA concentrations and changes in SCFA-producing bacteria and their metabolism (17, 21, 31, 32). In patients with brain metastases from NSCLC, total SCFA levels in feces were lower than in those with early-stage NSCLC and healthy controls, with particularly marked decreases in acetate and butyrate (21); whereas in post-operative NSCLC patients, those with higher prognostic nutritional indices exhibited an enrichment of SCFA-producing bacteria such as *Akkermansia* and *Eubacterium hallii*, accompanied by elevated butyrate/isovalerate levels and increased CD8^+^ T-cell infiltration (31). In the setting of immunotherapy, several clinical investigations indicate that variations in SCFAs and SCFA-producing bacteria are linked to ICIs treatment outcomes, primarily in NSCLC: NSCLC responders or those with long-term benefit often exhibit an expansion of butyrate-producing bacteria such as *Faecalibacterium*, accompanied by elevated levels of SCFAs including butyrate, propionate and acetate; higher SCFA levels correlate with longer progression-free survival (PFS), a lower risk of progression, and an improved response to anti-PD-1 therapy; notably, butyrate itself is positively correlated with PD-1 expression on circulating CD8^+^ and Vδ2^+^ T cells (32, 33).

Changes in Trp and its metabolites are among the alterations in metabolite profiles associated with the gut microbiota in NSCLC most closely linked to immune regulation. Clinical evidence has shown that plasma levels of Trp and xanthurenic acid are reduced in NSCLC patients, while levels of 3-hydroxyanthranilic acid (3-HAA)—a downstream product of kynurenine (Kyn), a Trp metabolite—are elevated (34). In NSCLC patients receiving ICI therapy, lower 3-HAA levels are associated with objective response and longer PFS (34), while higher Trp levels and lower levels of 3-hydroxykynurenine (3-HK), anthranilic acid and quinolinic acid are associated with better overall survival (OS) (35). In an NSCLC cohort receiving second-line nivolumab, Trp metabolites such as indole and its derivatives were suggested to play potential signaling roles in gut microbial functionality and may have implications for immune responses (36). Across clinical studies of advanced EGFR-mutated and early-stage NSCLC receiving immunotherapy, quinolinic acid was associated with treatment response in advanced disease, whereas quinolinic acid and oxindole-3-acetic acid were included as candidate metabolomic biomarkers of response in early-stage disease (37, 38).

With regard to bile acid metabolism, existing studies indicate that serum levels of primary bile acids (including cholic acid (CA), taurocholic acid (TCA) and glycocholic acid (GCA)) are elevated in NSCLC patients, whereas levels of the secondary bile acid lithocholic acid (LCA) are reduced (39). Furthermore, the gut microbiota of NSCLC patients and lung cancer-bearing mice exhibited reduced functional potential for SBAs metabolism (40). Notably, deoxycholic acid (DCA) levels are elevated in immunotherapy responders with EGFR-mutated NSCLC and in chemotherapy-sensitive lung cancer patients (37, 41), suggesting that SBA alterations may vary according to treatment context. In addition to classical bacterial metabolites, non-targeted fecal metabolomics studies have also revealed reduced levels of various aromatic and polyphenol-related metabolites in lung cancer patients; these metabolites are associated with bacterial communities such as *Ruminococcus gnavus*, *Lachnospira* and *Bacillota* (42).To address changes in main metabolites and their relationship to immunotherapy efficacy, representative clinical and translational studies are summarized in **Table 1**.

**Table 1:** Summary of key metabolite studies in NSCLC immunotherapy.

Metabolites	Population/cohort	Species	n	Sample type	Method	Treatment	Change	Immune endpoint	Main findings	Evidence	Ref.
SCFAs											
Propionate; butyrate	NSCLC cohort receiving second-line nivolumab	Human	11	Faeces	GC–MS/SPME; ¹H-NMR	Nivolumab (anti-PD-1)	SCFAs enriched in LRs; propionate ↑; butyrate NS	NR	SCFAs levels associated with long-term beneficial effects	HO	(158)
Acetate; propionate; butyrate	Stage III–IV NSCLC cohort	Human	22	Serum	GC–MS/MS	Anti-PD-1 + chemotherapy	Acetate/propionate/butyrate ↑ in responders	Circulating CD8^+^/Vδ2^+^ T-cell PD-1 expression correlated with SCFAs	Serum SCFAs levels may be a biomarker of anti-PD-1 efficacy.	HO	(33)
SCFAs	Stage III–IV NSCLC cohort	Human	55	Faeces	GC–MS	ICIs	No significant SCFA difference by response or irAE	irAE association NS	No predictive SCFA marker identified	HO	(159)
Butyrate; acetate; hexanoate	Advanced NSCLC cohort	Human	30	Faeces	GC–MS	ICB	Butyrate/acetate/hexanoate ↑ in responders	NR	SCFAs levels related to the efficacy of immunotherapy	HO	(17)
Total SCFAs; butyrate; propionate	Advanced NSCLC cohort	Human	26	Faeces	GC–MS	ICI + cisplatin-based chemotherapy	Total SCFAs, butyrate, and propionate ↑ with PFS ≥6 months	Butyrate positively correlated with peripheral lymphocyte and T-cell counts	SCFA levels associated with response to chemo-immunotherapy	HO	(111)
Bile acids											
TLCA; GCDCA	NSCLC cohort	Human	76	Plasma	Targeted UPLC–MS/MS (BA panel)	Anti-PD-1/PD-L1	GCDCA/TLCA ↑ associated with better PFS; TLCA ↑ in responders	NR	GCDCA and TLCA associated with better PFS; TLCA associated with ICI response	HO	(78)
TLCA	LLC1 syngeneic lung-tumor model	Mouse	—		Flow cytometry; tumor-volume analysis	TLCA administration ± anti-PD-1	—	cDC1, NK and CD8^+^ T cells ↑; effector/memory CD8^+^ T cells ↑	TLCA reduced tumor growth and potentiated anti-PD-1 efficacy	PM	
DCA	Advanced NSCLC cohort	Human	16	Faeces	Targeted UHPLC–MS/MS	First-line chemoimmunotherapy; antibiotic exposure	DCA ↓ after antibiotic exposure	NR	Antibiotic exposure associated with DCA ↓ and poorer chemoimmunotherapy outcomes	HO	(77)
	LLC tumor-bearing mice	Mouse	—	Serum	Untargeted/targeted UHPLC–MS/MS	Antibiotics ± paclitaxel + anti-PD-1; DCA supplementation	DCA ↓ with antibiotics	CD4^+^/CD8^+^ tumor infiltration ↓ after antibiotics	DCA supplementation improved chemoimmunotherapy efficacy and CD4^+^/CD8^+^infiltration	PM	
Trp-related											
Trp; xanthurenic acid; 3-HAA	Advanced/recurrent NSCLC cohort	Human	29	Plasma	LC–MS/MS	Nivolumab or pembrolizumab monotherapy	Trp/xanthurenic acid ↓ and 3-HAA ↑ vs healthy controls; 3-HAA ↓ in responders	NR	Low 3-HAA associated with longer PFS and better response prediction	HO	(34)
Trp; 3-HK; anthranilic acid; QA	Advanced/recurrent NSCLC cohort	Human	53	Plasma	HPLC–ESI–MS; LC–ESI–MS/MS	Nivolumab or pembrolizumab monotherapy	Higher Trp and lower 3-HK/anthranilic acid/QA associated with longer OS	PBMC transcriptome supported a lower-risk immune profile.	Plasma metabolite profile associated with OS (including Trp-related metabolites)	HO	(35)
IAA	NSCLC germ-free mice with CMT167/LLC validation models	Mouse	4	Faeces	Targeted LC–MS/MS; untargeted LC–MS	*B*. *animalis* gavage; oral IAA	IAA ↑ after *B. animalis* colonization	M2 macrophages ↓; CD8^+^/IFN-γ^+^ T cells ↑ in NSCLC models	*B. animalis*-derived IAA was linked to an AhR/METTL3/STAT3 mechanism and reduced NSCLC growth	PM	(93)
5-HIAA	NSCLC clinical cohort	Human	14 +21	Serum	ELISA	Anti-PD-1 mAb	5-HIAA ↑ in non-responders	NR	Higher serum 5-HIAA associated with anti-PD-1 failure	HO	(160)
	LLC tumor-bearing mice	Mouse	3–5			Anti-PD-1 mAb ± phenelzine and *A. muciniphila*	5-HIAA ↑ in NR-FMT; ↓ after phenelzine/*A. muciniphila*	CD8^+^ T-cell infiltration/function ↑ after 5-HIAA-lowering intervention	Lowering 5-HIAA accompanied better tumor control	PM	

Only NSCLC-related components that directly measured a metabolite and reported a metabolite change (including null findings) are retained. Human and NSCLC mouse components are separated. Population/model specifies the NSCLC cohort or model and the study system. n denotes the metabolite-analysis sample size; —, assay-specific n not stated.

Methods: GC–MS, gas chromatography–mass spectrometry; GC–MS/MS, tandem GC–MS; ¹H-NMR, proton nuclear magnetic resonance; UPLC–MS/MS, ultra-performance liquid chromatography–tandem MS; UHPLC–MS/MS, ultra-high-performance liquid chromatography–tandem MS; LC–MS/MS, liquid chromatography–tandem MS; HPLC–ESI–MS, high-performance liquid chromatography with electrospray-ionization MS; ELISA, enzyme-linked immunosorbent assay. “Targeted” indicates predefined metabolite quantification; “untargeted” indicates discovery metabolomics.

HO, human observational; PM, preclinical mouse model; NR, no directly corresponding immune endpoint; NS, not significant; ICI, immune checkpoint inhibitor; FMT, fecal microbiota transplantation; LLC, Lewis lung carcinoma; BA, bile acid; PFS/OS, progression-free/overall survival; TLCA, taurolithocholic acid; GCDCA, glycochenodeoxycholic acid; DCA, deoxycholic acid; IAA, indole-3-acetic acid; 5-HIAA, 5-hydroxyindoleacetic acid; 3-HAA, 3-hydroxyanthranilic acid; 3-HK, 3-hydroxykynurenine; QA, quinolinic acid. Evidence labels denote study type, not formal quality grading; LRs, long-term responders; ICB, immune checkpoint blockade; anti-PD-1 mAb, anti-programmed cell death 1 monoclonal antibody; NR-FMT, fecal microbiota transplantation from non-responders.

It is worth noting that there is no simple one-to-one correspondence between the gut microbiota and metabolites. Diverse microbial communities, which vary between individuals, can produce similar metabolite profiles (43), or genes involved in the synthesis of a particular metabolite may be distributed across different microbial communities (44); consequently, changes in the abundance of a single bacterial genus do not fully reflect the actual production of the corresponding metabolite. Cross-feeding between microbial communities also influences metabolite production; *in vitro* co-culture experiments using ¹³C-lactic acid tracing, Belenguer et al. demonstrated that *Eubacterium hallii* and *Anaerostipes caccae* converted lactate derived from *Bifidobacterium adolescentis* into butyrate (45). Furthermore, this metabolic network is regulated by external factors: different dietary substrates and luminal pH can alter the composition of SCFAs, while antibiotics may impair microbiota-mediated secondary bile acid transformation (43, 46, 47).

## 3 Research progress on the immunoregulatory functions of key gut microbiota-derived metabolites in NSCLC

Gut microbiota-derived metabolites serve as crucial molecular messengers linking the gut microbiota to the host immune system. In recent years, their roles have received increasing attention. These metabolites and precursors cannot only remotely shape the pulmonary immune microenvironment via the circulatory system but also directly influence the biological behavior of tumor cells. This section will systematically review the sources, target receptors, immunoregulatory mechanisms and bidirectional effects of these three groups on the etiology, progression and immunotherapy of lung cancer.

### 3.1 Direct microbiota-derived metabolites: short-chain fatty acids

SCFAs are major microbial metabolites produced through the fermentation of largely indigestible dietary carbohydrates by the human gut microbiota; they include acetate, propionate and butyrate, whose production involves multiple bacterial taxa, including *Bifidobacterium*, *Bacteroides* and *Faecalibacterium* (48–51). SCFAs can be absorbed from the gut into the peripheral circulation, where they exert systemic effects via circulating immune cells or bone marrow progenitor cells, thereby indirectly influencing the lungs (51, 52). However, human pharmacokinetic data show that the total SCFA concentration in the circulation decreases significantly from 375 ± 70 μmol/L in the portal vein to 79 ± 22 μmol/L in peripheral blood, and that only approximately 2% of colon-derived butyrate enters the systemic circulation, suggesting extensive utilization by the intestinal epithelium and robust first-pass clearance by the liver (51, 53). This concentration gradient is crucial for understanding the pulmonary immunoregulatory effects of SCFAs. Although *in vitro* functional experiments often utilize butyrate concentrations of 0.1–5 mmol/L, the concentration of butyrate in peripheral blood under physiological conditions is far lower than this. Consequently, the direct pharmacological effects of gut-derived SCFAs in the lungs may not depend on receptor saturation at high concentrations, but are more likely to occur indirectly via progressive epigenetic modifications (histone deacetylase (HDAC) inhibition) at low concentrations, or following the reprogramming of immune cell precursors in the gut or bone marrow, followed by cellular homing to the lungs. Although a small number of studies have demonstrated that pulmonary microbiota can synthesize SCFAs, the majority of data indicate that gut microbiota are the primary source of SCFAs (52, 54). Typically, SCFAs exert their effects via two mechanisms: firstly, by activating G protein-coupled receptors (GPCRs), such as free fatty acid receptor 3 (FFAR3/GPR41), free fatty acid receptor 2 (FFAR2/GPR43) and hydroxycarboxylic acid receptor 2 (HCAR2/GPR109A); secondly, by inhibiting HDACs, thereby altering the epigenetic state of cells (55, 56). Furthermore, GPR41 and GPR43 are present in alveolar macrophages, type II alveolar epithelial cells and airway epithelial cells (8, 57).

The immunoregulatory effects of SCFAs in lung cancer are distinctly bidirectional. Recent studies suggest that acetate tends to contribute to immune evasion in lung cancer, while butyrate exhibits anti-tumor effects. Wang et al. found that acetate is enriched in human NSCLC tissues, and that increased acetate uptake by tumor cells was demonstrated in mouse tumor models and human NSCLC cell lines (48). Mechanistic *in vitro* studies demonstrated that upon entering lung cancer cells, acetate promotes the acetylation of c-Myc (a transcription factor), thereby increasing its stability; this in turn induces elevated PD-L1 expression in tumor cells, as well as the expression of glycolytic enzymes, monocarboxylate transporter 1 (MCT1) and cell cycle-related genes (48). Preclinical co-culture experiments further showed that acetate-treated tumor cells inhibit CD8^+^ T-cell activation, thereby facilitating immune evasion (48). Furthermore, mechanistic studies using mouse-derived MDSCs and Ffar2-deficient models showed that acetate activates FFAR2 (GPR43) on the surface of myeloid-derived suppressor cells (MDSCs) within the lung cancer microenvironment, thereby initiating the Gαq/Ca²^+^/peroxisome proliferator-activated receptor-γ (PPAR-γ) signaling and upregulating arginase 1 (ARG1) expression (49). This increases the consumption of L-arginine, a key metabolic substrate required for T cells to maintain activation, proliferation and effector function, thereby impairing CD8^+^ T-cell function and creating an immunosuppressive cancer microenvironment. In a preclinical Lewis lung carcinoma (LLC) mouse model, FFAR2 inhibition reduced MDSC and ARG1 levels, increased tumor-infiltrating CD8^+^ T cells, and potentiated anti-PD-1 treatment (49).

Unlike acetate, butyrate has been shown to act on CD8^+^ T cells in experimental studies. In experiments using human CD8^+^ T cells, butyrate increased H3K27ac/H3K9ac levels in the PDCD1 and CD28 promoter regions of CD8^+^ T cells and promoted PD-1 and CD28 expression, thereby enhancing the T-cell receptor (TCR)/CD28 signaling pathway; this drives the production of anti-tumor cytokines, including interferon-γ (IFN-γ), tumor necrosis factor-α (TNF-α) and interleukin-2 (IL-2), and may enhance responsiveness to PD-1 blockade (33). It should be noted that although butyrate can enhance TCR signaling in CD8^+^ T cells and potentiate anti-PD-1 therapy, previous experimental studies have also indicated that, under specific conditions, butyrate may promote regulatory T-cell (Treg) expansion or affect treatment tolerance (50, 58). These findings suggest that the effects of butyrate may depend on its concentration, treatment context, target cell type and microbial source (59, 60). Bishoyi et al. noted that butyrate generally exerts a protective effect, but under some treatment settings it may be linked to multidrug resistance (61). Therefore, how to balance its beneficial effects against potential adverse impacts remains a key issue in translational applications. Furthermore, *in vitro* experiments by Thome et al. found that sodium butyrate exposure increases the expression of IFN-γ-R1 in peripheral blood CD4^+^ T cells, while simultaneously inhibiting the production of IFN-γ and granzyme B by CD8^+^ T cells and natural killer (NK) cells (62). Various studies have shown that 0.1–0.5 mmol/L butyrate promotes the differentiation of mouse-derived forkhead box P3 (Foxp3)^+^ Tregs, whereas 0.5 mmol/L enhances TCR signaling in human cytotoxic T cells; by contrast, 5 mmol/L suppresses IFN-γ and granzyme B expression in CD8^+^ T cells when tested in peripheral blood mononuclear cells (PBMCs) derived from NSCLC patients (33, 58, 62). By comparison, butyrate levels in normal human peripheral blood are typically only in the low micromolar range, which is lower than the concentrations used in most *in vitro* functional experiments. It therefore remains uncertain whether enterically derived butyrate can sustain effective concentrations in the lungs under physiological conditions; its distal effects may also be achieved indirectly via intestinal or bone marrow immune reprogramming (63).

Regarding the mechanism of action of butyrate, the literature often provides a generalized summary stating that ‘at low concentrations, it acts primarily as an HDAC inhibitor (64), while at high concentrations, it activates signaling via GPR41/43 (65)’. However, upon rigorous examination of its pharmacological dose–response relationship, this dichotomy is not strictly accurate. Receptor pharmacology studies have shown that the half-maximal effective concentration (EC_50_) values for butyrate activation of FFAR3/GPR41 and FFAR2/GPR43 are approximately 42–158 μmol/L and 28–371 μmol/L, respectively (66, 67), while *in vitro* HDAC activity assays its half-maximal inhibitory concentration (IC_50_) for HDAC inhibition is approximately 0.09 mmol/L (i.e. 90 μmol/L) (68). It is thus evident that there is significant overlap between the effective concentration ranges for butyrate’s activation of GPCRs and its inhibition of HDACs (both in the range of tens to hundreds of micromoles per litre), rather than the strict sequential or hierarchical order traditionally assumed. Consequently, within the *in vivo* microenvironment, the immunomodulatory effects of butyrate are not simply determined by a ‘concentration threshold’ triggering a switch in mechanism, but are more likely to be exerted synergistically via both the GPCR and HDAC pathways simultaneously within this overlapping concentration range. The ultimate biological outcome (pro-inflammatory or anti-inflammatory, pro-tumor or anti-tumor) depends on the target cell type (e.g. CD8^+^ T cells, Tregs or MDSCs), receptor expression levels, the integration status of downstream signaling pathways, and metabolic competition within the local microenvironment. These differential regulatory mechanisms indicate that the therapeutic use of SCFAs necessitates precise control of dosage as well as timing (**Figure 2**).

**Figure 2:**
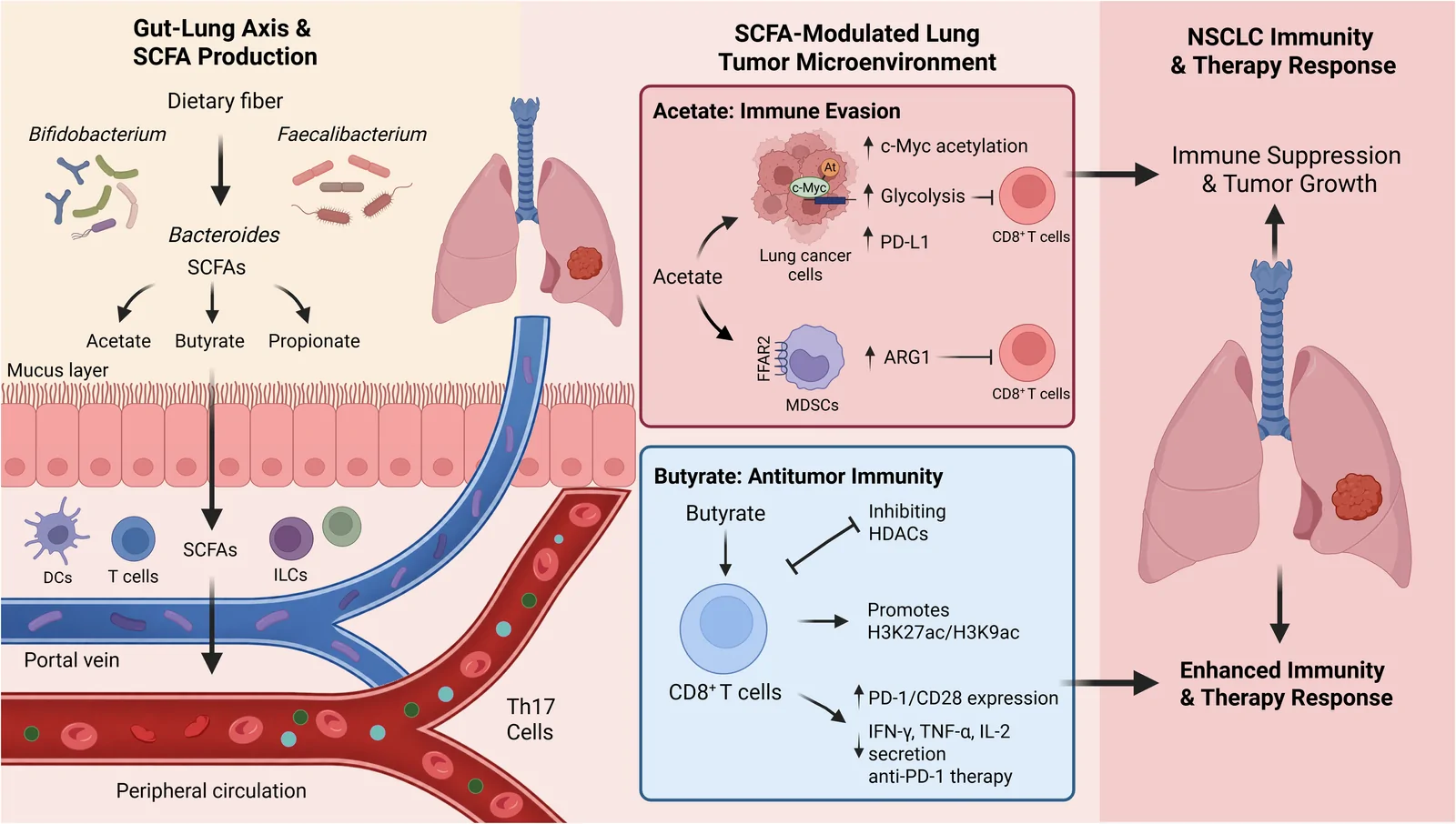
Gut-derived SCFAs and their bidirectional immunoregulation: gut microbiota ferment dietary fiber into acetate, propionate, and butyrate. After absorption, SCFAs undergo extensive first-pass clearance. SCFAs act via GPCRs (FFAR2/GPR43, FFAR3/GPR41, HCAR2/GPR109A) and HDAC inhibition. In NSCLC, acetate promotes immune evasion through c-Myc acetylation/PD-L1 upregulation, enhanced glycolysis/MCT1, and MDSC FFAR2–PPAR-γ–ARG1-mediated CD8^+^ T-cell suppression. Butyrate enhances CD8^+^ T-cell TCR/CD28 signaling, IFN-γ/TNF-α/IL-2 production, and anti-PD-1 responses, but its net effect is dose- and context-dependent. GPCR activation and HDAC inhibition overlap in effective concentration ranges, requiring precise dosing and timing. The acetate- and butyrate-mediated immune effects shown here are primarily supported by preclinical and ex vivo studies, whereas SCFA–treatment-response associations are derived from observational NSCLC cohorts. Created with BioRender.com.

### 3.2 Host–microbiota co-metabolites: secondary bile acids

SBAs are a class of microbiota-derived bile acids formed by the conversion of liver-derived primary bile acids by the gut microbiota. In a cohort of patients with NSCLC, the median serum concentrations of DCA and LCA were 152 and 14.9 nmol/L, respectively (39). In cell-based receptor assays, the EC_50_ values for G protein-coupled bile acid receptor 1 (TGR5/GPBAR1) were 1.01 μmol/L for DCA and 0.53 μmol/L for LCA; the corresponding values for farnesoid X receptor (FXR/NR1H4) were 47.31 and 34.90 μmol/L, respectively (69, 70). Given that circulating DCA and LCA concentrations in patients with NSCLC are below their EC_50_ values for TGR5 and FXR, we speculate that local accumulation of bile acids in the tumor microenvironment may increase their concentrations sufficiently to activate these receptors. However, this possibility remains unconfirmed, as direct measurements of DCA and LCA concentrations in human NSCLC tumor tissue are lacking. Hepatic primary bile acids in humans include CA and chenodeoxycholic acid (CDCA). After their conjugation to glycine or taurine and subsequent biliary delivery to the intestine, gut bacterial enzymes catalyse deconjugation, 7α-dehydroxylation, oxidation and epimerization, thereby converting them into the representative secondary bile acids DCA and LCA (71–73). In addition to participating in lipid digestion, bile acids also function as signaling molecules on receptors such as FXR (NR1H4) and TGR5 (GPBAR1), contributing to host metabolic homeostasis, intestinal mucosal defense, and the regulation of inflammatory and immune responses (74). FXR is present across multiple lung cell types, including alveolar epithelial cells, alveolar macrophages and lung fibroblasts (75, 76). DCA and LCA can activate alveolar epithelial cells and pulmonary fibroblasts via FXR (76).

Clinical evidence is gradually establishing a link between bile acid metabolic disorders and immune dysregulation in NSCLC. In a cohort of 387 patients with advanced NSCLC, antibiotic exposure was associated with decreased efficacy of first-line chemoimmunotherapy, accompanied by disturbances in bile acid metabolism and the gut microbiota (77). Lee et al. conducted a metabolomic analysis of 76 NSCLC patients receiving anti-PD-1/PD-L1 monotherapy and found that levels of the conjugated secondary bile acid taurolithocholic acid (TLCA) and the conjugated primary bile acid glycochenodeoxycholic acid (GCDCA) were associated with longer PFS, with TLCA levels being higher in responders (78). These studies suggest an association between gut microbiota-associated bile acid metabolism and immunotherapy efficacy in NSCLC. Feng et al. further found that gut microbiota metabolic functional modules (GMMs) associated with SBAs were reduced in the fecal microbiota of NSCLC patients and in lung cancer-bearing mice. In the mouse model, the proportion of CD4^+^Foxp3^+^CD25^+^ Treg cells in the spleens increased, while the proportions of CD3^+^ T cells, CD4^+^ T cells and the CD4^+^/CD8^+^ ratio decreased (40), suggesting that disrupted bile acid metabolism may have immunosuppressive effects.

At the mechanistic level, bile acid-related immunoregulation in NSCLC may involve two receptor pathways: TGR5 (GPBAR1) and FXR (NR1H4). TGR5, a bile acid membrane receptor expressed by macrophages, promotes the polarization of tumor-associated macrophages (TAMs) towards an M2-like immunosuppressive phenotype (79). Early studies found that TGR5 is abnormally overexpressed in NSCLC tissues, is associated with later clinical stages and poorer prognosis, and has been shown in preclinical models to promote tumor cell proliferation, migration and invasion via the Janus kinase 2 (JAK2)/signal transducer and activator of transcription 3 (STAT3) signaling pathway (80). In LLC-bearing mouse and mouse-derived bone marrow-derived macrophage models, TGR5 deficiency inhibits the polarization of TAMs towards an M2-like pro-tumor phenotype, thereby lifting the suppression of CD8^+^ T cells by TAMs. TGR5 promotes the expression of M2-associated molecules such as ARG1 and interleukin-10 (IL-10) via cyclic adenosine monophosphate (cAMP)–STAT3/STAT6 signaling (STAT6, signal transducer and activator of transcription 6), thereby attenuating anti-tumor immunity; elevating cAMP levels can partially restore the M2 phenotype in Tgr5-deficient macrophages (79). FXR, as a nuclear bile acid receptor, shows a negative correlation with PD-L1 among patients with NSCLC and can identify an FXR^high^/PD-L1_low_ NSCLC subgroup (81), suggesting its potential as an immune-related biomarker in NSCLC. Herpesvirus entry mediator (HVEM) shows a positive correlation with FXR and an inverse correlation with PD-L1 in NSCLC patient tissues (82). Preclinical experiments further showed that FXR can directly transcriptionally activate TNFRSF14 (which encodes HVEM), or activate the protein kinase B (Akt), extracellular signal-regulated kinase 1/2 (ERK1/2) and STAT3 signaling pathways to promote G1/S cell cycle progression, thereby upregulating HVEM expression. HVEM inhibits the function of CD8^+^ T cells and NK cells via the B and T lymphocyte attenuator (BTLA) pathway, while simultaneously increasing immunosuppressive elements, including Tregs and M2-like TAMs. Anti-BTLA therapy can partially restore anti-tumor immunity and inhibit the growth of tumors with high FXR expression (82).

Furthermore, in splenic T cells isolated from C57BL/6 and OT-1 mice, the conjugated secondary bile acid TLCA promotes T-cell proliferation, upregulates molecules associated with T-cell activation and cytotoxic function—such as IFNG (interferon-γ) and TNF (tumor necrosis factor)—and enhances tumor cell killing mediated by OT-1 CD8^+^ T cells; in MC38 and LLC1 syngeneic mouse tumor models, TLCA treatment increases the proportion of conventional type 1 dendritic cells (cDC1), NK cells and CD8^+^ T cells in tumors and draining lymph nodes, and produces a synergistic anti-tumor effect with anti-PD-1 therapy (78).

### 3.3 Microbiota-regulated host tryptophan metabolism and microbial indole derivatives

Trp is an essential aromatic amino acid that the human body cannot synthesize endogenously; beyond its involvement in protein synthesis, it serves as a substrate for both host cells and the gut microbiota to produce various derivatives with immunomodulatory activity (83, 84). Trp can enter three major metabolic pathways—kynurenine, 5-hydroxytryptamine and indole—with the involvement of host cells and gut microbiota (83). In mammals, Trp primarily enters the kynurenine pathway via indoleamine 2,3-dioxygenase 1/2 (IDO1/IDO2) or tryptophan 2,3-dioxygenase 2 (TDO2), producing Kyn, which is further metabolized to 3-HK, 3-HAA and quinolinic acid, among other products (85). The gut microbiota, on the other hand, can directly convert Trp into indole and its derivatives, such as indole-3-acetic acid (IAA), indole-3-propionic acid (IPA), indole-3-lactic acid (ILA) and indole-3-aldehyde (IAld); several of these products act as aryl hydrocarbon receptor (AhR) ligands, regulating the host immune response in a ligand- and cell-type-dependent manner (84, 86–88). Furthermore, the gut microbiota can directly or indirectly regulate the host’s kynurenine and 5-hydroxytryptamine metabolic pathways (89). IDO1 is expressed in NSCLC tumor cells, stromal cells and dendritic cells, while TDO2 may also play a role in Kyn generation in certain LUADs and in the context of treatment resistance (90, 91).

Quantitative measurements provide clinical context for these metabolic pathways. A pre-treatment NSCLC cohort showed mean plasma concentrations of 46.8 ± 17.8 μmol/L for Trp, 2.448 ± 0.716 μmol/L for Kyn and 1.996 ± 1.771 μmol/L for IAA (35). In cell-based NSCLC studies, Kyn was applied at 100 μmol/L and increased AhR nuclear accumulation and IDO1 activity (92), whereas IAA was used at 10 ng/mL (approximately 0.057 μmol/L) (93). These concentrations were specific to the respective experimental systems and should not be interpreted as physiological activation thresholds. Direct measurements of Kyn or IAA in NSCLC tumor tissue remain limited (**Figure 3**).

**Figure 3:**
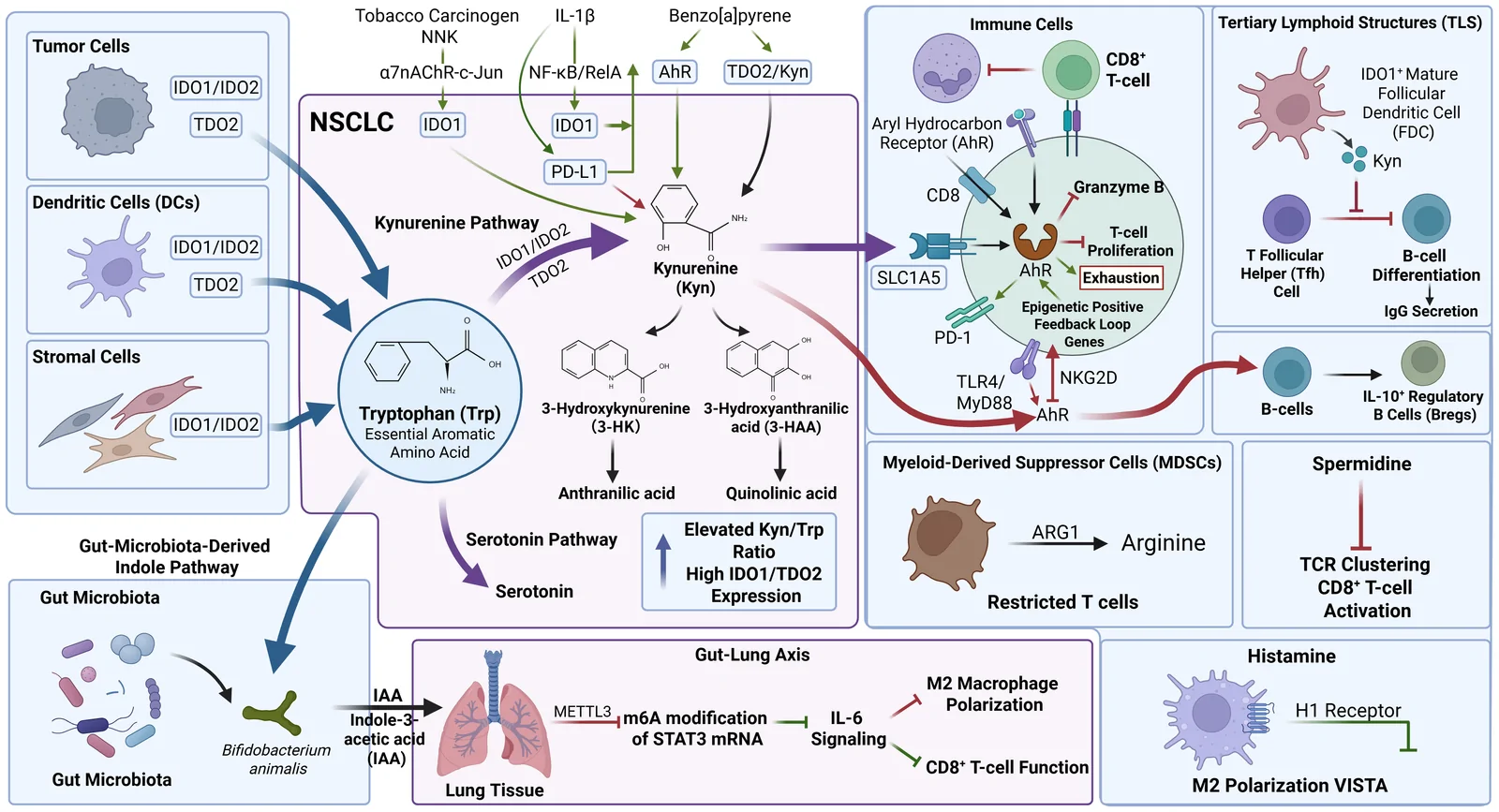
Gut microbiota-associated tryptophan metabolism and other amino acid derivatives: Trp is an essential dietary amino acid metabolized via the kynurenine, 5-hydroxytryptamine, and indole pathways. In NSCLC, IDO1/TDO2-driven Kyn production activates AhR, depletes Trp, promotes CD8^+^ T-cell exhaustion, downregulates NKG2D, expands Tregs/Bregs/MDSCs, and upregulates PD-L1, contributing to immunosuppression and ICI resistance. Kyn/Trp, Trp, 3-HAA, 3-HK, and tumor IDO1 expression correlate with ICI response; FMT plus pembrolizumab responders show reduced Kyn/quinolinic acid. Gut microbiota-derived indoles (IAA, IPA, ILA, IAld) act as AhR ligands; *B. animalis*-derived IAA inhibits METTL3/m^6^A-STAT3/IL-6 signaling, reducing M2 macrophages and enhancing CD8^+^ T cells. Other derivatives: ARG1-mediated arginine depletion, spermidine-induced TCR inhibition/MDSC expansion, and histamine-H1R-driven M2/VISTA immunosuppression. Clinical associations involving Kyn/Trp-related metabolites are observational, whereas the immune mechanisms and *B. animalis*-derived IAA effects are supported mainly by preclinical studies. Created with BioRender.com.

#### 3.3.1 The IDO1/TDO2–Kyn pathway

Reshaping of the Trp–kynurenine pathway may correlate with responsiveness to ICIs in NSCLC. Botticelli et al. found that a higher Kyn/Trp ratio prior to treatment was associated with early progression on nivolumab and shorter PFS and OS in patients with NSCLC (86), suggesting that enhanced Trp catabolism may be linked to primary resistance to anti-PD-1 therapy. Kocher et al., however, found that patients with metastatic NSCLC treated with nivolumab or pembrolizumab who achieved disease control had significantly higher baseline Trp levels than those with primary resistance; receiver operating characteristic (ROC) analysis showed that a baseline Trp level of ≥49.3 μmol/L was associated with disease control in this cohort, with a sensitivity of 88.9% and a specificity of 71.4%; however, there were no significant differences in Kyn levels or the Kyn/Trp ratio (87), suggesting that the potential predictive value of different Trp metabolic markers may not be consistent and requires further validation. As described in Section 2.2, several downstream Trp metabolites, including 3-HAA, 3-HK, anthranilic acid and quinolinic acid, are associated with ICI outcomes in NSCLC (34, 35). Smoking status may also be associated with Trp–Kyn metabolism and its association with ICI outcomes. Liang et al. reported lower Trp levels, higher Kyn levels and a lower Trp/Kyn ratio in smoking patients with NSCLC than in non-smokers. Among smoking patients receiving anti-PD-1 therapy, a lower Trp/Kyn ratio and higher tumor IDO1 expression were associated with poorer disease control (88).

Of note, tumor tissue IDO1 expression differs from metabolic markers in significance. Bessede et al. analyzed the transcriptomes of 891 NSCLC tumors and found that higher IDO1 expression correlated with improvements in the objective response, PFS and OS; however, this association was observed only in the atezolizumab treatment group, not in the docetaxel group (94), while experimental findings also suggested an immunosuppressive role for IDO1-derived L-Kyn (94). Therefore, high IDO1 expression may serve as a marker of pre-existing anti-tumor immunity, or it may induce negative-feedback immune suppression via Kyn. Corresponding changes in the Trp pathway have also been documented in studies involving gut microbiota intervention. Furthermore, in a Phase II clinical trial, 20 patients with NSCLC received fecal microbiota transplantation (FMT) plus first-line pembrolizumab treatment. Clinical analyses showed that responders had lower Kyn and quinolinic acid levels and reduced fecal Trp pathway activity than non-responders; higher plasma quinolinic acid was also associated with reduced effector memory CD8^+^ T cells and increased Tregs in patient PBMCs (95).

At the functional and mechanistic level, environmental carcinogens and inflammatory signals can promote the activation of IDO1/TDO2 in NSCLC. The tobacco carcinogen 4-(methylnitrosamino)-1-(3-pyridyl)-1-butanone (NNK) drives IDO1 upregulation through the α7 nicotinic acetylcholine receptor (α7nAChR)–c-Jun axis (88); interleukin-1β (IL-1β) induces IDO1 via the nuclear factor-κB (NF-κB)/RelA pathway and PD-L1 expression (96); benzo[a] pyrene (BaP), on the other hand, directly activates AhR or induces TDO2 to generate endogenous Kyn, thereby enhancing AhR signaling and upregulating PD-L1 and inducible T-cell co-stimulator ligand (ICOSL) (90). Activated IDO1 disrupts immune surveillance via a dual pathway: on the one hand, it depletes Trp, thereby restricting Trp availability (96); on the other hand, accumulated Kyn induces T-cell exhaustion by activating AhR in CD8^+^ T cells, upregulating PD-1 and downregulating granzyme B (97). Zhu et al. further revealed, using LUAD mouse models and ex vivo CD8^+^ T-cell experiments, that solute carrier family 1 member 5 (SLC1A5) mediates the Kyn uptake by CD8^+^ T cells; Kyn–AhR signaling subsequently epigenetically activates SLC1A5 and immune checkpoint gene loci, forming a positive feedback loop of ‘increased uptake—exacerbated exhaustion’ (98). Furthermore, Kyn can downregulate NK group 2 member D (NKG2D) expression on the surface of NK and CD8^+^ T cells (91), and promote the formation of IL-10^+^ regulatory B cells (Bregs) via the Toll-like receptor 4 (TLR4)–myeloid differentiation primary response 88 (MyD88) and AhR pathways (99), thereby contributing to a broader immunosuppressive network. Moreover, in cisplatin-resistant human NSCLC cells, exogenous Kyn also promoted AhR nuclear accumulation and increased IDO1 activity and LAT1 expression, suggesting that Kyn–AhR signaling can act directly on tumor cells (92).

Furthermore, IDO1 is also expressed on mature follicular dendritic cells within the TLS of NSCLC; in an ex vivo coculture assay using human tonsil-derived T follicular helper (Tfh) cells and memory B cells, L-Kyn inhibited Tfh-mediated IgG secretion, suggesting that this pathway may limit local anti-tumor immunity within the TLS (94). It is worth noting that high IDO1 expression is commonly observed in inflammatory-type NSCLC with high immune cell infiltration; this may be the result of an active immune response, but elevated IDO1 may, in turn, inhibit T-cell and B-cell function through the production of Kyn, thereby forming an immunosuppressive negative feedback loop. The Kyn pathway may also be associated with tolerance to ICIs. In platinum-resistant NSCLC mouse models, dual inhibition of IDO1 and TDO2 reduces Kyn-related immunosuppression, enhances NKG2D expression on NK cells and CD8^+^ T cells, and reduces Tregs and MDSCs, thus improving the antitumor efficacy of anti-PD-1 therapy (91). In preclinical radioresistant NSCLC models, PRMT3 suppresses CD8^+^ T-cell function by upregulating IDO1 and increasing Kyn production, while PRMT3 inhibition enhances the therapeutic effect of radiotherapy combined with anti-PD-L1 therapy (97).

#### 3.3.2 Indole derivatives and other amino acid metabolites from the gut microbiota

The anti-tumor potential of indole derivatives derived from the gut microbiota has also attracted attention. Song et al. reported a significant depletion of *Bifidobacterium animalis in* fecal samples from NSCLC cases; preclinical studies further showed that the addition of this bacterium or its key metabolite IAA can activate AhR in lung tissue through the gut–lung axis, downregulate methyltransferase-like 3 (METTL3) expression and reduce N^6^-methyladenosine (m^6^A) modification of STAT3 transcripts, thereby inhibiting interleukin-6 (IL-6) signaling, reducing M2-type macrophage polarization and enhancing CD8^+^ T-cell function, thus inhibiting NSCLC progression (93).

In addition to Trp and its metabolites, other amino acid derivatives are also involved in tumor immune regulation. Certain gut bacteria can produce polyamines, including putrescine and spermidine (100, 101), and convert histidine into histamine (102). Polyamines such as spermidine, which are downstream of ornithine, can also inhibit TCR aggregation and CD8^+^ T cell activation in mouse tumor models, while driving MDSC expansion (103). Furthermore, *in vivo* and *in vitro* experiments demonstrated that histamine, a histidine derivative, can promote M2-like polarization of macrophages and the expression of the inhibitory immune checkpoint molecule V-domain Ig suppressor of T-cell activation (VISTA) by activating histamine H1 receptors on their surface, thereby causing T-cell dysfunction and diminishing responsiveness to ICIs (104).

### 3.4 Metabolite network regulation

SCFAs, SBAs and Trp derivatives may interact at multiple levels—including gut microbiota metabolism, the intestinal barrier and host immune signaling—to form a dynamic regulatory network. Firstly, SCFAs and SBAs interact in the regulation of intestinal barrier function. In a mouse model of *Clostridium difficile* infection, butyrate treatment increased fecal levels of SBAs such as DCA and LCA, increased the abundance of bile salt hydrolase (BSH)-producing bacteria, and enhanced intestinal FXR expression; inhibiting BSH or FXR, however, attenuates the barrier-protective and anti-inflammatory effects of butyrate, suggesting that SCFAs regulate the intestinal barrier by influencing bile acid conversion and FXR signaling (105). Conversely, in C57BL mice, long-term excessive exposure to DCA can lead to microbial dysbiosis and reduced intestinal FXR activity, while intestinal-selective FXR agonists can alleviate mucosal damage and increase the levels of SCFA-producing bacteria (106). Furthermore, butyrate can increase the recruitment of AhR to the cytochrome P450 family 1 subfamily A member 1 (CYP1A1) promoter by inhibiting HDACs, thereby enhancing the AhR transcriptional effects of Trp-derived ligands such as 6-formylindolo[3,2-b]carbazole (FICZ) and IAA; moreover, the interaction between butyrate and FICZ has been validated in human colonic tissue (107). Furthermore, in experimental studies showed that Trp depletion can enhance Kyn uptake (108), AhR expression and promote Treg differentiation, while butyrate can also promote Treg formation by modulating epigenetic regulation (50), suggesting that these two pathways may converge at the immune endpoint of Treg differentiation. However, validation of these metabolic interactions in animal models of NSCLC and patient tumor tissues is still lacking; future studies should utilize simultaneous quantitative analysis of multiple metabolites and combinatorial interventions to verify the direction, intensity and overall immunological effects of these interactions within the lung cancer microenvironment.

In summary, SCFAs, SBAs and Trp derivatives synergistically shape the immune microenvironment of lung cancer through mechanisms such as metabolic competition, epigenetic reprogramming and nuclear receptor/membrane receptor signaling crosstalk, respectively. The present study reveals the high heterogeneity and condition-dependence of metabolite functions—metabolites within the same class (e.g. acetic acid and butyric acid among SCFAs, and DCA and TLCA among SBAs) may exert diametrically opposed immunomodulatory effects. This complexity suggests that future translational research should focus on: ① establishing NSCLC immunological subtyping based on metabolite profiles; ② elucidating the mechanisms underlying the dynamic functional switching of specific metabolites at different stages of tumor progression; ③ exploring new strategies for synergistically sensitising ICIs by simultaneously targeting multiple metabolic pathways (e.g. concurrent inhibition of IDO1 and supplementation with butyrate-producing bacteria). Precise regulation of the gut microbiota metabolite network promises to be a new breakthrough in overcoming resistance to NSCLC immunotherapy.

## 4 Clinical translational research on gut microbiota-derived metabolites in NSCLC

### 4.1 Early screening and staging assessment

The gut microbiota and its associated metabolites have shown potential for lung cancer detection and risk stratification, although most clinical evidence has been derived from NSCLC cohorts. Zheng et al. analyzed gut microbiota in feces from 42 patients with early-stage NSCLC and 65 healthy controls, and constructed a support vector machine model based on 13 differentially expressed operational taxonomic units (OTUs); the AUCs in the discovery cohort and independent validation cohort were 0.976 and 0.764, respectively (28). Zhao et al. further conducted a combined analysis of fecal microbiota and serum metabolomics, finding that the AUC for individual metabolites such as lysophosphatidylethanolamine (LysoPE) 18:3 and lysophosphatidylcholine (LysoPC) 14:0 was approximately 0.89–0.91, and that these were significantly correlated with lung cancer-associated microbiota such as *Erysipelotrichaceae* UCG-003 and *Clostridium* (109). An analysis of the fecal metabolome in 52 NSCLC patients revealed that L-cystathionine levels were reduced in patients with stage I–II disease, while phthalic acid and L-lactic acid levels were elevated in those with stage III–IV disease; phthalic acid levels were further elevated in advanced-stage patients compared with those in earlier stages, suggesting that the levels of specific metabolites are significantly associated with lung cancer staging (24). Zeng et al. conducted Mendelian randomization analyses of overall lung cancer and its major subtypes, including LUAD, lung squamous cell carcinoma and SCLC, and identified 13 gut microbial taxa and 15 plasma metabolites causally associated with overall lung cancer risk. Further mediation analyses suggested that *Alloprevotella*, *Ruminococcaceae* UCG-003 and *Collinsella* may be linked to lung cancer risk through circulating metabolites including docosapentaenoic acid (DPA), γ-glutamylvaline and serine, respectively (110). These findings identify gut microbiota-derived metabolites as candidate risk-associated biomarkers, although their diagnostic and prognostic utility requires further clinical validation.

### 4.2 Strategies for enhancing NSCLC immunotherapy based on gut microbiota-derived metabolites and metabolic pathways

Considering the relationship between gut microbiota-derived metabolites and the response to NSCLC immunotherapy, current translational research is primarily being conducted along three pathways: firstly, the direct supplementation of metabolites with immunomodulatory activity or the targeting of key enzymes in their metabolic pathways; secondly, the holistic remodeling of the gut microbiota through FMT; and thirdly, the regulation of microbial metabolic functions through dietary interventions and probiotics. These three approaches complement one another at the molecular, community and lifestyle levels, collectively providing a translational framework for investigating strategies to optimize ICI treatment based on gut microbiota-derived metabolites (**Figure 4**).

**Figure 4:**
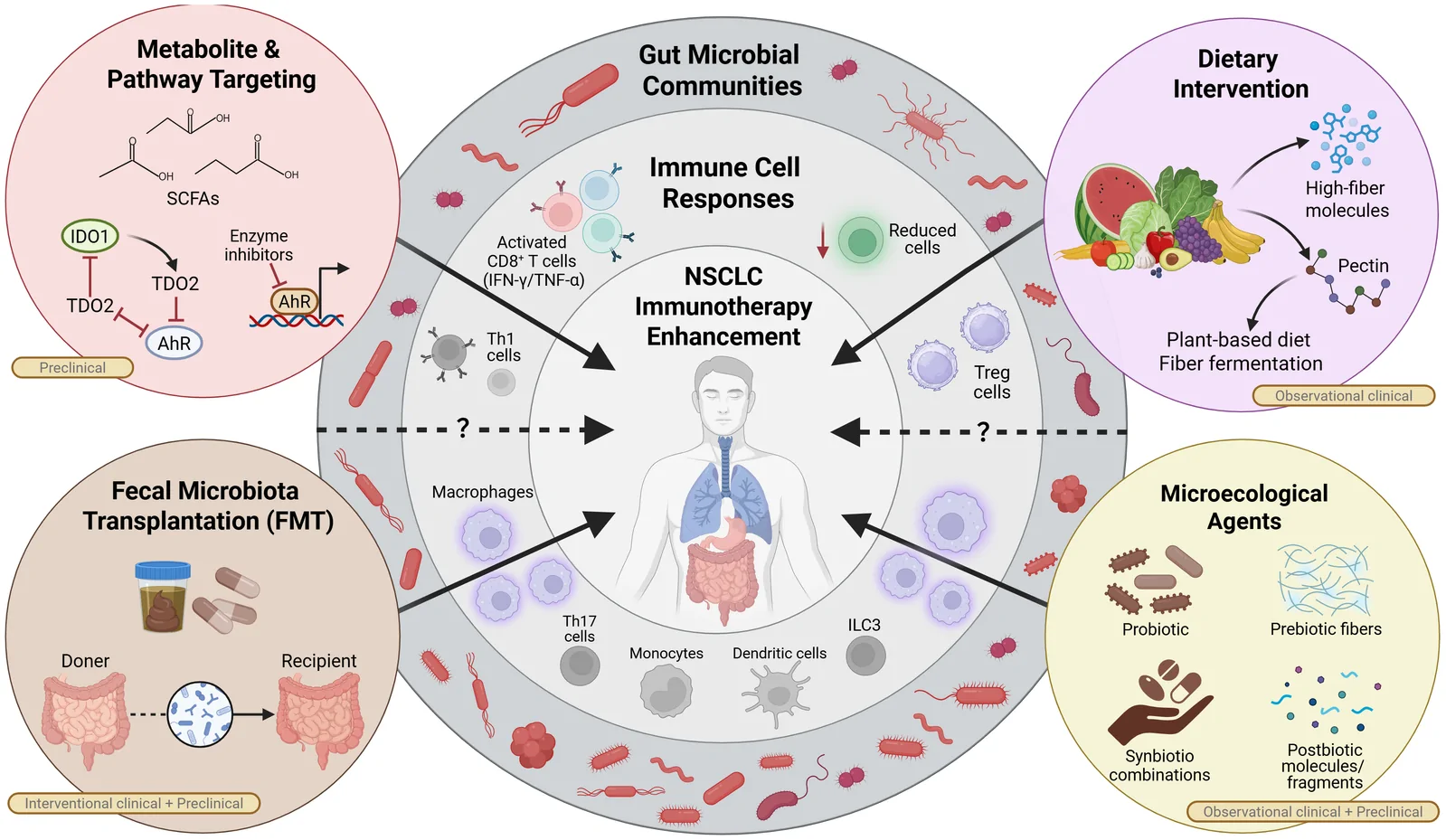
Major strategies for enhancing NSCLC immunotherapy based on gut microbiota-derived metabolites and metabolic pathways: Four complementary approaches are illustrated: (1) Metabolite & pathway targeting—SCFAs and inhibitors targeting IDO1/TDO2 or AhR; (2) Fecal microbiota transplantation (FMT)—transfer of donor fecal microbiota to recipients; (3) Dietary intervention—plant-based diet, high-fiber molecules, and pectin promote fiber fermentation; and (4) Microecological agents—probiotics, prebiotic fibers, synbiotic combinations, and postbiotic molecules/fragments. Created with BioRender.com.

#### 4.2.1 Supplementation with microbial metabolites and targeting of metabolic pathways

Direct supplementation with defined microbiota-derived metabolites that possess immunomodulatory activity represents a potential strategy for enhancing immunotherapy in NSCLC; preclinical evidence regarding SCFAs is relatively consistent, but human interventional evidence is limited. Clinical evidence indicates that, among NSCLC patients receiving immunotherapy, responders show a greater abundance of SCFAs in their feces or serum (17, 33). Preclinical studies provide mechanistic support for these associations, although not all models are NSCLC-specific. In B16-F0 melanoma-bearing mice, butyrate combined with anti-PD-1 therapy significantly inhibited tumor growth and prolonged survival; the CD8^+^ T-cell-dependent mechanism is described in Section 3.1 (33). Yang et al. further found that in antibiotic-treated LLC mice, oral supplementation with an SCFA mixture comprising sodium acetate, sodium propionate and sodium butyrate enhanced the tumor-suppressive effects of the combination of pemetrexed, cisplatin and anti-PD-1 therapy, increased the infiltration of CD45^+^, CD3^+^ and CD8^+^ T cells within the tumor, and up-regulated the expression of granzyme B and TNF-α in CD8^+^ T cells (111). As described in Section 3.3.2, *B. animalis*-derived IAA acts through the gut–lung axis to modulate antitumor immunity. Oral IAA supplementation inhibited tumor growth in orthotopic CMT167 and subcutaneous LLC lung cancer models (93). Furthermore, in multiple non-NSCLC mouse tumor models, gut-derived inosine can drive T-helper 1 (Th1) responses through adenosine A_2_A receptors on T cells and enhance ICI responsiveness when co-stimulatory signaling is sufficient (112). This suggests that the translational value of these metabolites in NSCLC requires further investigation.

In addition to direct metabolite supplementation, pharmacological targeting of metabolic enzymes and sensing receptors associated with gut microbiota-regulated metabolites may also offer potential strategies for NSCLC immunotherapy. Among these, the Trp–Kyn pathway has received considerable attention. Early clinical studies of the IDO1 inhibitor epacadostat combined with pembrolizumab, with or without chemotherapy, showed preliminary antitumor activity in patients with advanced NSCLC (113, 114). However, these studies had small sample sizes and lacked concurrent control groups, making it difficult to determine whether epacadostat provided an additional therapeutic benefit. Subsequent randomized Phase II trials failed to demonstrate additional clinical benefit from adding epacadostat to pembrolizumab-based regimens (115, 116). These findings indicate that the IDO1 inhibition strategies evaluated did not provide a demonstrable additional clinical benefit in the regimens tested, potentially owing to inadequate target inhibition and metabolic pathway compensation. In the ECHO-305/KEYNOTE-654 trial, epacadostat at 100 mg twice daily reduced circulating Kyn levels without normalising them, suggesting that pathway inhibition may have been incomplete (115). Furthermore, in a preclinical platinum-resistant NSCLC model, compensatory TDO2 upregulation was observed following IDO1 inhibition, providing a rationale for dual-target intervention (91). The downstream receptor AhR has also been explored as a therapeutic target, with its selective inhibitor BAY 2416964 showing preliminary target-modulating activity and acceptable tolerability in an early-phase clinical trial (117). These pharmacological findings may inform future strategies targeting microbiota-associated Trp–Kyn–AhR signaling, although the efficacy of microbiota-directed interventions remains to be established. Regarding HDAC-targeted strategies, butyrate, a gut microbiota-derived SCFA, can act as a natural HDAC inhibitor. However, synthetic HDAC inhibitors often target multiple HDAC subtypes and may cause adverse effects (118). More selective agents, such as TNG260 targeting the HDAC1-containing CoREST complex, are under investigation, but their clinical efficacy remains uncertain (119). Nevertheless, the pharmacological effects of synthetic HDAC inhibitors cannot be directly equated with those of microbiota-derived butyrate (**Figure 5**).

**Figure 5:**
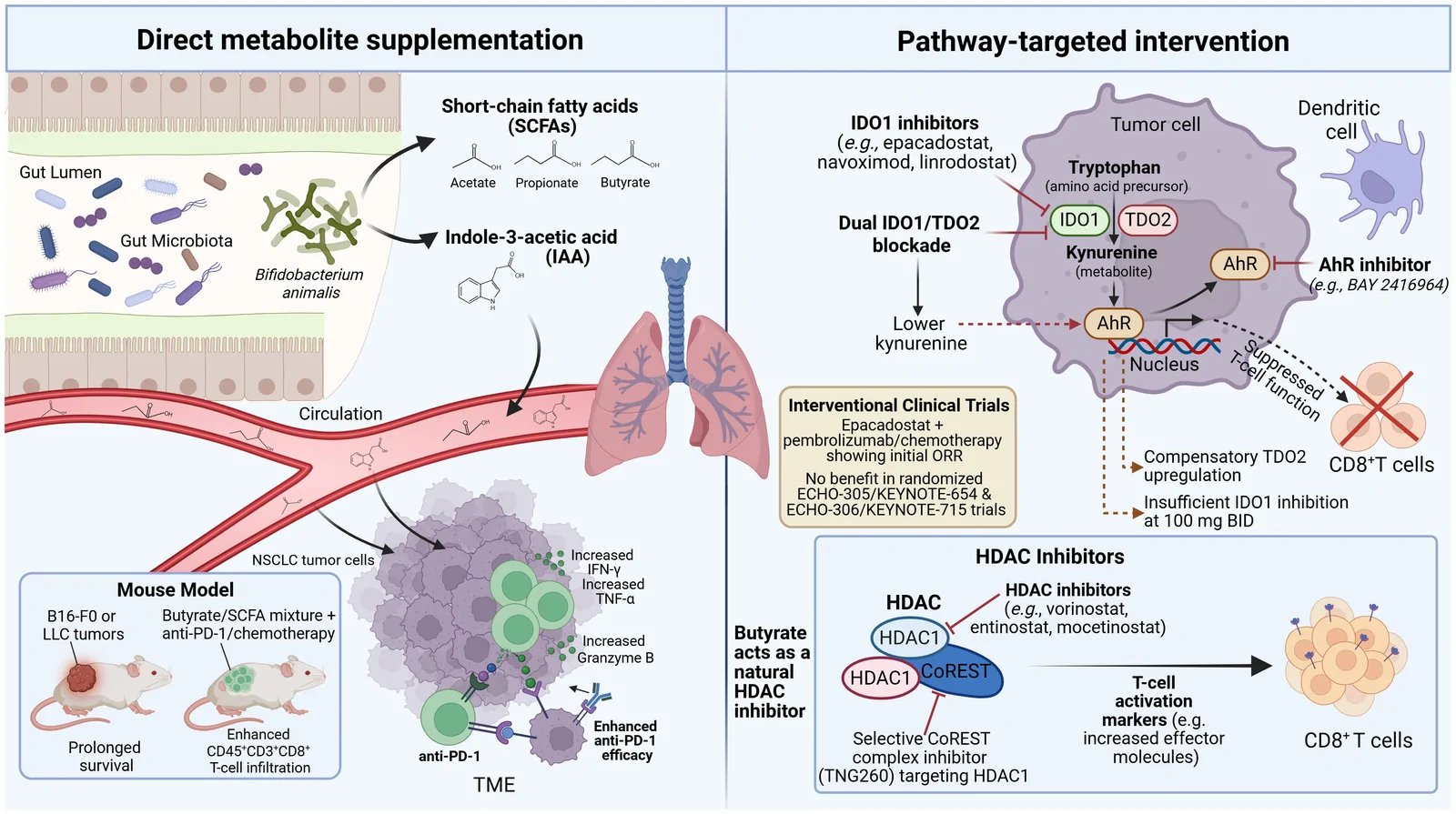
Microbiota-associated kynurenine metabolites and pathway targeting NSCLC immunotherapy: SCFAs are enriched in immunotherapy responders. Butyrate plus anti-PD-1 inhibits tumor growth via CD8^+^ T cells, and an SCFA mixture improves chemo-immunotherapy in LLC mice. *B. animalis*-derived IAA improves antitumor immunity in NSCLC models. Targeting the Trp–Kyn pathway: single-agent IDO1 inhibitors show limited clinical benefit; dual IDO1/TDO2 inhibition or AhR blockade may overcome pathway compensation. HDAC inhibitors combined with PD-L1 blockade show modest activity; subtype/CoREST-selective TNG260 is under investigation. Created with BioRender.com.

#### 4.2.2 Fecal microbiota transplantation

FMT is a therapeutic procedure whereby the fecal microbiota obtained from screened donors is processed and transplanted into the recipient’s gastrointestinal tract to modulate or restore the gut microbiota of the recipient (120). At present, the established clinical application of FMT is primarily focused on recurrent or refractory *Clostridioides difficile* infections (121), while its use in anti-tumor immunotherapy remains at an exploratory stage. Unlike traditional indications, FMT donors in tumor immunotherapy studies may be sourced from either healthy individuals or responders to ICI therapy (95, 122–124). In melanoma, FMT combined with anti-PD-1 therapy has demonstrated preliminary efficacy in salvage therapy following resistance and in first-line treatment (122, 124). Recently, a growing body of research has begun to explore the use of FMT in NSCLC immunotherapy (see **Table 2** for a summary). In a multicenter, single-arm Phase II trial, 20 treatment-naïve patients with advanced NSCLC and a PD-L1 tumor proportion score (TPS) of ≥50% received a single oral dose of FMT derived from healthy donors in combination with pembrolizumab. The results showed an ORR of 80% (16/20, all partial responses) and a disease control rate of 95%, with no treatment-related adverse events of grade 3 or above (95). However, the small sample size, single-arm design, and lack of a randomized control group mean that patient selection and assessment biases cannot be excluded, and the respective contributions of FMT and pembrolizumab cannot be distinguished; therefore, these findings warrant cautious interpretation. Metagenomic analysis revealed that responders and non-responders did not differ significantly with respect to donor strain engraftment, while responders exhibited a more pronounced clearance of harmful gut microbiota at baseline. Analysis across NSCLC and melanoma cohorts further revealed that the cleared bacterial species primarily included *Enterocloster citroniae* and other microbiota previously associated with ICI resistance (95). This treatment was also associated with alterations in Trp metabolism as discussed in Section 3.3.1 (95). In addition to first-line treatment, FMT has also begun to be used for retreatment following resistance to immunotherapy. Huang et al. administered oral FMT in combination with a PD-1 inhibitor to 27 patients with advanced NSCLC who had developed secondary PD-1 resistance; 20 of these patients also received concurrent chemotherapy. The analysis reported an ORR of 29.6% and a disease control rate of 63.0%, with median PFS reaching 6.8 months (125). Responders exhibited higher gut microbiota diversity, accompanied by elevated peripheral CD8^+^ T cell numbers and a reduction in Tregs, and no FMT-related serious infections occurred (125). Furthermore, studies have indicated that in antibiotic-pretreated LLC mice, FMT derived from responders can improve the tumor-suppressing efficacy of chemotherapy combined with anti-PD-1 therapy (111). Overall, FMT can treat diseases by regulating gut microbial homeostasis and immunity via microorganisms and their metabolites (126); existing research suggests the potential for FMT in the immunotherapy of NSCLC and indicates that gut microbiota metabolites and metabolite precursors such as SCFAs and Trp may be involved in its immunomodulatory effects. However, there is significant inter-individual variability in FMT engraftment efficiency. In patients with anti-PD-1-resistant melanoma, Baruch et al. divided 10 patients into two groups to receive FMT from two different donors who had responded to immunotherapy. The findings indicated a shift in microbial composition from the baseline across all patients; however, the three patients who achieved objective remission all came from the same donor group, while no remissions were observed in the other donor group (122); Davar et al. included 15 evaluable patients, of whom 6 derived clinical benefit, with their microbiota clearly shifting towards that of the corresponding donor; among the remaining 9 patients who did not benefit, only about half exhibited similar changes (123). This may be influenced by factors such as the degree of matching between the donor’s and recipient’s baseline microbiota, the colonization resistance of the recipient’s original microbiota, and exposure to antibiotics (127). Currently, evidence for FMT in NSCLC primarily consists of small-sample, single-arm trials, which can only be used for preliminary assessments of safety and feasibility. Randomized controlled trials, by establishing concurrent controls, can more reliably assess whether FMT produces an independent benefit on top of the same immunotherapy regimen. Furthermore, there is currently no standardization regarding donor selection, preparation and administration protocols for FMT, and its long-term safety requires further validation through randomized controlled trials.

**Table 2:** Published evidence and registered trials of fecal microbiota transplantation relevant to NSCLC immunotherapy.

ID	Status	Phase	Design	Population	Enrollment	Intervention	Key results/primary endpoints	Ref.
Initial treatment/sensitization								
NCT04951583	Unknown	II	Multicentre; single-arm	Advanced NSCLC; PD-L1 TPS ≥50%	20	Single oral healthy-donor FMT before first-line pembrolizumab	ORR 80%; DCR 95%; 1-year PFS 65%; no grade ≥3 FMT/ICI-related AEs	(95)
ChiCTR2300076829	NR	II	Prospective; single-arm	Stage IIIB–IV LUAD; PD-L1 <1%	13 enrolled; 10 evaluated	Oral healthy-donor FMT + first-line pembrolizumab/platinum chemotherapy	DCR 60%; 12-month PFS 55% vs 28% in matched external controls (HR 0.36); no grade ≥3 FMT-related AEs	(161)
NCT07247786 (MIGRANT)	Not yet recruiting	II	Randomised; open-label	Resectable stage II–III NSCLC	68	Responder-derived oral FMT + neoadjuvant durvalumab/paclitaxel/carboplatin vs no FMT	pCR	—
NCT04105270	Recruiting	II	Randomised; double-blind	Untreated advanced LUAD	82	Oral RMT vs placebo, both with durvalumab/platinum/pemetrexed	PFS and safety	—
NCT04924374 (MORELIA)	Completed	—	Randomised; open-label	ICI-naïve NSCLC	25	Oral FMT + ICI vs no FMT	FMT safety	—
NCT05008861	Unknown	I	Single-arm	Untreated advanced NSCLC	20	Washed FMT capsules + anti-PD-(L)1/platinum chemotherapy	FMT/ICI-related AEs	—
NCT06403111 (FMT-JSNO-02)	Unknown	II	Single-arm	Untreated driver-negative advanced NSCLC; PD-L1 <50%	62	Oral FMT + tislelizumab/platinum chemotherapy	12-month PFS	—
ICI resistance/rechallenge								
ChiCTR2100043472	NR	NR	Prospective; single-arm	Advanced NSCLC after ICI progression	7	Repeated oral healthy-donor FMT + camrelizumab rechallenge	ORR 14.3%; DCR 28.6%; mPFS 1.5 months; mOS 12.1 months; FMT-related AEs were grade 1	(162)
MR-36-22-010054	NR	II	Single-arm	Stage IIIB–IV NSCLC; acquired PD-1 resistance	27	Oral FMT + PD-1 rechallenge; 20/27 also received chemotherapy	ORR 29.6%; DCR 63.0%; mPFS 6.8 months; mOS 11.1 months; no serious FMT-related infections	(125)
NCT04521075	Unknown	I/II	Single-arm	Mixed cancers including NSCLC after PD-(L)1 progression	42 total (2 NSCLC planned)	ICI-responder-derived FMT + nivolumab	FMT-related AEs and ORR	—
NCT05286294 (MITRIC)	Active, not recruiting	II	Single-arm	Mixed cancers including NSCLC after ICI progression	20	ICI-responder-derived FMT + standard ICI	safety and ORR	—
NCT05669846	Recruiting	II	Single-arm	Stage IV NSCLC; PD-L1 ≥1%; PD-(L)1 progression	26	Healthy-donor FMT + pembrolizumab	ORR	—
NCT07001618 (IMMUNOLIFE2)	Recruiting	II	Randomised; open-label	Advanced NSCLC; recent antibiotics; progression within 6 months	162	Oral MaaT033 + cemiplimab vs investigator-choice chemotherapy	12-week DCR	—
NCT07432984 (FMT-LUNG)	Not yet recruiting	II	Single-arm	Advanced NSCLC after chemotherapy and ICI failure	15	Colonoscopic FMT from NSCLC responders or healthy donors + tislelizumab	safety	—
IrAEs management								
NR	—	—	Case series	LUAD and pulmonary sarcomatoid tumor; refractory ICI colitis	5 total (1 LUAD)	Colonoscopic FMT for refractory atezolizumab- or pembrolizumab-associated colitis	LUAD: remission after two FMTs. Sarcomatoid tumor: transient improvement, later requiring ileostomy.	(163)
NR	—	—	Case series	Stage IIIA LUAD with refractory durvalumab-associated colitis	2 total (1 LUAD)	Two colonoscopic FMTs for refractory durvalumab-associated colitis	Colitis improved to grade 1	(164)

Status denotes registry recruitment status (checked 15 October 2026). For published studies, N refers to the reported cohort.—, not applicable; NR, not reported. Abbreviations: AE, adverse event; DCR, disease control rate; FMT, fecal microbiota transplantation; HR, hazard ratio; ICI, immune checkpoint inhibitor; irAE, immune-related adverse event; LUAD, lung adenocarcinoma; mOS/mPFS, median OS/PFS; NSCLC, non-small cell lung cancer; ORR, objective response rate; OS, overall survival; pCR, pathological complete response; PD-1, programmed cell death protein 1; PD-L1, programmed death-ligand 1; PFS, progression-free survival; RMT, rational microbiome therapy; TPS, tumor proportion score.

#### 4.2.3 Dietary modulation and microbiome intervention strategies

Dietary intervention is attracting attention as a potential strategy for modulating gut microbiota-derived metabolites. A recent systematic review suggested that plant-based dietary patterns may be associated with gut microbial changes favourable to ICI efficacy, although direct clinical evidence in NSCLC remains limited (128). A prospective investigation into advanced NSCLC suggested that patients who encountered hyperprogressive disease (HPD) following ICI therapy had a poorer macronutrient balance in their diet, primarily characterized by fat intake exceeding recommended levels, and a tendency towards higher fat and dairy product consumption (129), suggesting that dietary patterns may be linked to the gut microbiota and ICI treatment outcomes. Fermentable dietary fibers such as inulin and pectin can modulate the gut microbiota and increase levels of SCFA metabolites in preclinical models, thus enhancing anti-PD-1 efficacy (130, 131). Furthermore, certain natural compounds with microbiota-modulating activity have also demonstrated potential to sensitize tumors to immunotherapy. In LLC mice, oral administration of ginseng polysaccharides combined with anti-PD-1 therapy augmented tumor suppression, while simultaneously increasing plasma levels of SCFAs (such as valerate) and reducing L-Kyn as well as the Kyn/Trp ratio (132). Castalagin, a polyphenolic prebiotic-like substance, enriches bacterial communities such as *Ruminococcaceae* and *Alistipes*, increases taurine-conjugated bile acids, and restores the tumor-suppressive effect of anti-PD-1 therapy in mice transplanted with the gut microbiota of patients with ICI-resistant NSCLC (133). Overall, the available evidence is derived mainly from observational clinical studies and preclinical experiments, and requires further validation in prospective randomized controlled trials.

Probiotic preparations are formulations that modulate the host microbiome by supplementing functional microorganisms or promoting their growth; they primarily include probiotics, prebiotics, postbiotics and synbiotics, and offer advantages such as relatively controllable composition, convenient administration and ease of standardization. In murine colorectal cancer models, supplementation with the probiotic strain *Lacticaseibacillus rhamnosus* Probio-M9 modulated gut microbial and metabolite profiles and enhanced anti-PD-1-mediated tumor control (134). Clinically, retrospective studies have reported associations between probiotic supplementation and improved responses to ICIs or prolonged PFS in patients with advanced or recurrent NSCLC receiving anti-PD-1 therapy (135, 136). A recent study indicates that this effect may vary depending on antibiotic exposure and the duration of probiotic use. Among antibiotic-treated patients, probiotics were associated with restoration of NSCLC immunotherapy efficacy regardless of the duration of use; whereas in patients not receiving antibiotics, probiotic use must exceed 15 days to improve immunotherapy efficacy (137). However, prospective randomized evidence demonstrating a causal benefit is lacking. Active metabolites produced by probiotics may serve as key mediators in regulating anti-tumor immunity in lung cancer. As described in Section 3.3.2, IAA produced by *B. animalis* improves anti-tumor immunity in an NSCLC model (93); cis-Bac429, derived from *Bacteroides*, promotes IFN-γ production by primary CD8^+^ T cells and makes anti-PD-1 treatment more effective in a mouse lung cancer model (138). Non-digestible dietary carbohydrates like resistant starch, inulin, or arabinogalactan are known as prebiotics, which can be fermented by the colonic microbiota to increase SCFA output (139). They also promote the growth of butyrate-producing bacteria, including *Ruminococcaceae* and *Faecalibacterium* (140). Postbiotics consist of inactivated microorganisms and/or their components; compared with probiotics containing live bacteria, postbiotics are characterized by relatively stable composition and storage, as well as a lower risk of infection, and may therefore be more suitable for cancer patients with compromised immune function. Studies have shown that the postbiotic JK5G can improve quality of life as well as nutritional status in patients with advanced NSCLC following immunotherapy and lower the rate of certain irAEs, including anemia and appetite loss; it also increases the concentrations of *Faecalibacterium*, *Ruminococcaceae* and butyrate in feces, while reducing *Escherichia–Shigella* relative abundance and simultaneously improving the tumor immune microenvironment (141). The postbiotic formulation MS-20 contains abundant microbial metabolites and is produced through fermentation of a soya-based medium by a consortium of probiotic bacteria and yeasts. Animal studies have demonstrated that MS-20 can change the composition of gut microbiota, increase the abundance of *Ruminococcus bromii*, boost the number of effector CD8^+^ T cells, and enhance the tumor-suppressing effects of anti-PD-1 against lung and colon cancers (142). Synbiotics are formulations combining probiotics with prebiotics; studies in mice with pulmonary inflammation have shown that *Bifidobacterium breve* M16-V, in combination with oligosaccharides and pectin, can improve lung function and reduce neutrophil-mediated inflammation, accompanied by elevated levels of SCFAs in feces (143). At present, evidence backing the application of synbiotics for lung cancer immunotherapy remains limited, although relevant clinical evaluations have already commenced. Furthermore, nano-encapsulation has been proposed to enhance the stability, gastrointestinal survival rate and delivery efficiency of synbiotics; materials such as starch, chitosan and nano-cellulose are considered promising carrier materials and may provide new formulation approaches for the application of synbiotics in lung cancer immunotherapy (144).

In summary, direct metabolite supplementation and pathway targeting offer a high degree of controllability at the molecular level, but are limited by the magnitude of effects of individual metabolites and pathway compensation; FMT can simultaneously reshape multiple metabolic functions at the community level, but donor variability and standardization challenges limit its reproducibility; diet and probiotic preparations offer advantages in terms of safety and accessibility, but their effects are mild and dependent on patient compliance. Future translational research may require a combination of the above strategies. For example, a baseline microbial–metabolic homeostasis could be established through FMT or dietary intervention, followed by precision enhancement using specific metabolites or pathway-targeting drugs, thereby achieving synergistic effects through ‘community remodeling + molecular targeting’.

## 5 Current controversies and limitations

Current techniques primarily employ fecal metagenomic sequencing combined with plasma or fecal metabolomics; the data obtained mostly reflect microbial abundance and metabolite concentrations at specific time points. It is difficult to determine the specific sources of metabolites, nor is it possible to directly prove that they are produced in the gut and subsequently reach the lungs or tumor tissue. Firstly, while stable isotope tracing can track the flow of metabolites, it is currently used primarily in animal experiments, and it is difficult to carry out continuous, multi-site sampling in NSCLC patients. Furthermore, feces do not fully represent the gut mucosal microbiota; fecal metabolites primarily reflect the residual metabolic pool that has not been absorbed or utilized, and are not equivalent to the actual exposure levels in the gut mucosa or the dose entering the circulation. Utilization by the intestinal epithelium, first-pass metabolism in the liver, and tissue uptake further contribute to differences in concentrations in the portal vein, peripheral blood and lung tissue. Lung tissue and bronchoalveolar lavage fluid (BALF) are low-microbial-load samples that are susceptible to background contamination from the oropharyngeal microbiota, sampling instruments and reagents; furthermore, the degree of dilution of the lavage fluid can also affect metabolite quantification (145–147). These factors limit paired analyses between the gut, circulation, lungs and tumor tissue. Secondly, existing studies are predominantly small-sample or cross-sectional in design, restricting the identification of causality; differences in microbial colonization, immunity and metabolism between animal models and humans also limit the accuracy of extrapolating results. Furthermore, the absence of standardized protocols for sample processing, sequencing platforms and data analysis hampers cross-study comparison and limits reproducibility. Future research should integrate multiple techniques, such as multi-site sampling, targeted metabolic profiling and stable isotope tracing, and conduct independent cohort validation in accordance with standardized reporting guidelines.

Gut microbiota-derived metabolites exert dual effects that remain a major point of contention in current research; these effects vary with concentration and microenvironmental context, and hence are difficult to classify as either beneficial or deleterious. Butyrate can boost the function of CD8^+^ T cells and improve the effectiveness of anti-PD-1 therapy; however, higher levels of circulating butyrate and propionate have also been linked to resistance to anti-CTLA-4 treatment and an increase in Tregs (33, 148). These observations suggest a potential therapeutic window for SCFA intervention, within which SCFAs may promote CD8^+^ T-cell activation or tumor suppression without inducing Treg expansion or suppressing effector cells at higher concentrations. However, clinically effective doses cannot yet be determined from *in vitro* experiments alone. Subsequent studies should determine the dosing range by combining pharmacokinetic data from blood and lung tumor tissues with cell-specific pharmacodynamic indicators. Consequently, combined strategies involving gut microbiota-derived metabolites and immunotherapy cannot be discussed in general terms without reference to specific treatment regimens. Differences also exist among Trp metabolites: Kyn can promote immunosuppression via AhR, while IAA derived from *B. animalis* exhibits antitumor activity in NSCLC models (93, 149). This indicates that the ultimate effects exerted by gut microbiota-derived metabolites are affected by various factors, including their type, concentration, site of action and treatment regimen. The clinical significance of the same microbiota is not entirely consistent across different studies. *Prevotella* has been reported as a pro-inflammatory bacterium in some NSCLC studies, while in others it has been associated with a better response to NSCLC immunotherapy (15, 150). This inconsistency may stem from the fact that immune effects are highly context dependent: long-term pro-inflammatory effects may promote tumor-associated inflammation and immune dysregulation, whereas, under PD-1 blockade, a higher state of immune activation may also be associated with disease control. An association between *Akkermansia*-related taxa and favorable treatment outcomes (151) or less severe irAEs (152) has been observed in multiple NSCLC cohorts. However, Haberman et al. found that this association is not limited to patients receiving ICI (153); Derosa et al. also demonstrated that the predictive power of a single bacterial species is limited, and that the relationship between *Akkermansia* and treatment response may not be a simple linear one. It therefore remains unclear whether *Akkermansia* genuinely and directly enhances ICI efficacy or merely reflects better overall health status in patients (154); resolving this issue requires functional evidence: observing changes in ICI efficacy following the single colonization of *Akkermansia* in germ-free mice or antibiotic-depletion models, or comparing the effects of targeted elimination of this bacterium against different backgrounds of *Akkermansia* abundance. Correlational data from observational cohorts alone are insufficient to elevate *Akkermansia* from a prognostic marker to a therapeutic target.

Several potential confounders should be considered when interpreting observational associations in NSCLC. These include treatment regimen, antibiotic and PPI exposure, dietary composition, smoking status, disease stage, histological subtype, EGFR status, geographical and ethnic background, and other host-related factors. Antibiotic exposure has been directly associated with poorer chemoimmunotherapy outcomes and alterations in gut microbiota and bile acid metabolism (77). PPI use has also been associated with poorer ICI outcomes, and one NSCLC study reported differences in fecal microbial profiles according to PPI exposure (155, 156). Dietary composition can influence the gut microbiota (46), and a prospective NSCLC cohort reported that higher fat and dairy intake was associated with hyperprogressive disease during ICI treatment (129). Smoking has been linked to altered tryptophan metabolism, increased IDO1 expression and differences in anti-PD-1 response in NSCLC (88). Disease stage, histological subtype and EGFR status have been associated with differences in gut microbial composition in NSCLC cohorts (24, 26, 27). Geographic and ethnic background may contribute to differences between cohorts, but current lung cancer studies provide mainly descriptive rather than causal evidence for this effect (16, 129). Failure to measure or adjust for these variables may result in residual confounding, and observational studies should clearly report which covariates were included in their analyses.

Furthermore, regarding whether gut-derived metabolites can reach the lungs and exert effects at functional concentrations, the existing evidence can be categorized into three levels. The first level involves isotope tracing to directly track carbon flux: Lund et al. fed mice with ¹³C-labeled dietary fiber and demonstrated that carbon produced by microbial fermentation can enter the metabolic pool of colonic epithelial cells and be incorporated into histone acetylation, providing direct tracer evidence for ‘microbiota–host carbon flux’; however, this study did not analyse lung tissue and tracked total carbon flow rather than the complete fate of specific metabolite molecules (157). The second level involves strain-specific functional knockout experiments that indirectly support the gut-to-lung transport of metabolites: following colonization of germ-free mice with a *Bacteroides* mutant strain deficient in propionate synthesis, propionate levels in lung tissue were significantly lower than in mice colonized with the wild-type strain, supporting systemic transport of gut-derived propionate to the lung (8); Song et al., through combined metabolomic analysis of *Bifidobacterium* culture supernatants and feces, serum and lung tissue from germ-free mice, identified IAA as a key candidate metabolite and validated IAA-mediated AhR signaling in the lung and its anti-tumor effects both *in vivo* and *in vitro*, suggesting that this metabolite not only reaches the lungs but is also sufficient to exert a functional effect (93). At the third level, human evidence remains largely correlational. In NSCLC and broader lung-cancer cohorts, SCFA and Trp-metabolite profiles have been associated with gut microbiota composition and immunotherapy response (32, 34, 35), but site-specific concentration measurements and causal validation remain limited. Overall, the existing evidence supports the notion that gut-derived metabolites can reach the lungs, but ‘reaching’ does not equate to ‘attaining a functional concentration’: with the exception of a few metabolites that can be actively taken up or locally concentrated in lung tissue, the concentrations of most metabolites in lung tissue may remain below the threshold for receptor activation. Whether their immunological effects result from direct action on pulmonary immune cells, indirect transmission via circulating immune cells, or primarily depend on local immune conditioning by the intestinal mucosa remains to be distinguished through multi-site paired sampling and tracer studies.

Overall, translational research into gut microbiota-derived metabolites in NSCLC immunotherapy faces dual methodological and biological challenges. Technically, existing detection methods struggle to accurately track the gut-to-lung flow of metabolites, while limitations in sample acquisition and a lack of standardization further restrict cross-site paired analyses and the reproducibility of results. Biologically, the immunomodulatory effects of metabolites are concentration-, microenvironment- and treatment-regimen-dependent, and the clinical significance of the same bacterial species varies across different cohorts, suggesting that stable and specific gut microbiota predictive biomarkers are still lacking. Future research must focus on multi-site dynamic sampling, stable isotope tracing and functional validation experiments, combined with standardized reporting protocols and validation in independent cohorts, in order to advance this field from descriptive association studies to the stage of causal validation.

## 6 Conclusions and outlook

In conclusion, gut microbiota-derived metabolites function as crucial mediators that bridge the gut microbiome and the immune microenvironment of NSCLC. SCFAs, gut microbiota-associated Trp metabolites and bile acid metabolites, among others, can influence immune cell function, tumor immune evasion and the effectiveness of ICIs through the gut–lung axis. Therefore, supplementation with beneficial metabolites, dietary and probiotic interventions, FMT, and targeting metabolic enzymes or receptors are being explored as candidate adjunctive strategies for NSCLC immunotherapy.

Future work will need to adopt standardized longitudinal multi-omics designs with paired, multisite sampling of stool, portal and peripheral blood, lung tissue, bronchoalveolar lavage fluid and tumor tissue, where feasible. Stable-isotope and metabolic-flux tracing can help define gut-to-lung metabolite transfer, tissue exposure and functional concentrations, whereas germ-free or antibiotic-depleted models can provide causal functional validation. Independent prospective cohorts are required to test candidate biomarkers, and randomized controlled trials should evaluate FMT, probiotics, dietary interventions and metabolite supplementation. Harmonised protocols for sample collection, metabolite quantification, sequencing, statistical analysis and reporting will be essential for improving reproducibility. Finally, dedicated studies of SCLC are needed because current evidence is overwhelmingly derived from NSCLC and remains insufficient for this subtype.

## Glossary

3-HAA: 3-hydroxyanthranilic acid
3-HK: 3-hydroxykynurenine
5-HIAA: 5-hydroxyindoleacetic acid
AE: Adverse event
AhR: Aryl hydrocarbon receptor
Akt: Protein kinase B
ARG1: Arginase 1
AUC: Area under the receiver operating characteristic curve
BA: Bile acid
BALF: Bronchoalveolar lavage fluid
BaP: Benzo[a] pyrene
Breg: Regulatory B cell
BSH: Bile salt hydrolase
BTLA: B and T lymphocyte attenuator
CA: Cholic acid
cAMP: Cyclic adenosine monophosphate
CD28: Cluster of differentiation 28
cDC1: Conventional type 1 dendritic cell
CDCA: Chenodeoxycholic acid
CoREST: Corepressor for RE1-silencing transcription factor
CR: Complete response
CTLA-4: Cytotoxic T-lymphocyte-associated protein 4
CYP1A1: Cytochrome P450 family 1 subfamily A member 1
DCA: Deoxycholic acid
DCB: Durable clinical benefit
DCR: Disease control rate
DPA: Docosapentaenoic acid
EC₅₀: Half-maximal effective concentration
EGFR: Epidermal growth factor receptor
ERK1/2: Extracellular signal-regulated kinase 1/2
F/B: *Firmicutes/Bacteroidetes* ratio (*Bacillota/Bacteroidota* under updated nomenclature)
FFAR2/GPR43: Free fatty acid receptor 2
FFAR3/GPR41: Free fatty acid receptor 3
FICZ: 6-formylindolo [3,2-b] carbazole
FMT: Fecal microbiota transplantation
Foxp3: Forkhead box P3
FXR/NR1H4: Farnesoid X receptor
GCA: Glycocholic acid
GCDCA: Glycochenodeoxycholic acid
GMM: Gut microbiota metabolic functional module
GPCR: G protein-coupled receptor
H1R: Histamine H1 receptor
H3K27ac: Histone H3 lysine 27 acetylation
H3K9ac: Histone H3 lysine 9 acetylation
HCAR2/GPR109A: Hydroxycarboxylic acid receptor 2
HDAC: Histone deacetylase
HDAC1: Histone deacetylase 1
HPD: Hyperprogressive disease
HR: Hazard ratio
HVEM: Herpesvirus entry mediator
IAA: Indole-3-acetic acid
IAld: Indole-3-aldehyde
ICI: Immune checkpoint inhibitor
ICOSL: Inducible T-cell co-stimulator ligand
IC₅₀: Half-maximal inhibitory concentration
IDO1: Indoleamine 2,3-dioxygenase 1
IDO2: Indoleamine 2,3-dioxygenase 2
IFN-γ: Interferon-γ
IFN-γR1: Interferon-γ
IFNG: Interferon gamma (gene)
IgG: Immunoglobulin G
IL-10: Interleukin-10
IL-1β: Interleukin-1β
IL-2: Interleukin-2
IL-6: Interleukin-6
ILA: Indole-3-lactic acid
IPA: Indole-3-propionic acid
irAE: Immune-related adverse event
JAK2: Janus kinase 2
Kyn: Kynurenine
LAT1: L-type amino acid transporter 1
LCA: Lithocholic acid
LLC: Lewis lung carcinoma
LUAD: Lung adenocarcinoma
LysoPC: Lysophosphatidylcholine
LysoPE: Lysophosphatidylethanolamine
MCT1: Monocarboxylate transporter 1
MDSC: Myeloid-derived suppressor cell
METTL3: Methyltransferase-like 3
MyD88: Myeloid differentiation primary response 88
m⁶A: N⁶-methyladenosine
NF-κB: Nuclear factor-κB
NK: Natural killer
NKG2D: Natural killer group 2 member D
NNK: 4-(methylnitrosamino)-1-(3-pyridyl)-1-butanone
NR: Not reported
NSCLC: Non-small cell lung cancer
ORR: Objective response rate
OS: Overall survival
OTU: Operational taxonomic unit
PBMC: Peripheral blood mononuclear cell
pCR: Pathological complete response
PD-1: Programmed cell death protein 1
PD-L1: Programmed death-ligand 1
PDCD1: Programmed cell death 1 (gene)
PFS: Progression-free survival
PPAR-γ: Peroxisome proliferator-activated receptor-γ
PPI: Proton pump inhibitor
PR: Partial response
PRMT3: Protein arginine methyltransferase 3
RMT: Rational microbiome therapy
ROC: Receiver operating characteristic
SBA: Secondary bile acid
SCFA: Short-chain fatty acid
SCLC: Small cell lung cancer
SD: Stable disease
SLC1A5: Solute carrier family 1 member 5
STAT3: Signal transducer and activator of transcription 3
STAT6: Signal transducer and activator of transcription 6
STK11: Serine/threonine kinase 11
TAM: Tumor-associated macrophage
TCA: Taurocholic acid
TCR: T-cell receptor
TDO2: Tryptophan 2,3-dioxygenase 2
Tfh: T follicular helper cell
TGR5/GPBAR1: Takeda G protein-coupled receptor 5
Th1: Type 1 T-helper cell
TLCA: Taurolithocholic acid
TLR4: Toll-like receptor 4
TLS: Tertiary lymphoid structure
TNF: Tumor necrosis factor (gene)
TNF-α: Tumor necrosis factor-α
TNFRSF14: Tumor necrosis factor receptor superfamily member 14
TNM: Tumor-node-metastasis
TPS: Tumor proportion score
Treg: Regulatory T cell
Trp: Tryptophan
VISTA: V-domain immunoglobulin suppressor of T-cell activation
α7nAChR: α7 nicotinic acetylcholine receptor
